# Functionalized Polymeric Micelles for Targeted Cancer Therapy: Steps from Conceptualization to Clinical Trials

**DOI:** 10.3390/pharmaceutics16081047

**Published:** 2024-08-06

**Authors:** Ana Serras, Célia Faustino, Lídia Pinheiro

**Affiliations:** Research Institute for Medicines (iMed.ULisboa), Faculdade de Farmácia, Universidade de Lisboa (ULisboa), Avenida Professor Gama PintoGama Pinto, 1649-003 Lisboa, Portugal; anafiserras@hotmail.com (A.S.); lpinheiro@ff.ulisboa.pt (L.P.)

**Keywords:** cancer, nanomedicine, polymeric micelles, amphiphilic polymers, targeted drug delivery, stimuli-responsive micelles, clinical trials

## Abstract

Cancer is still ranked among the top three causes of death in the 30- to 69-year-old age group in most countries and carries considerable societal and macroeconomic costs that differ depending on the cancer type, geography, and patient gender. Despite advances in several pharmacological approaches, the lack of stability and specificity, dose-related toxicity, and limited bioavailability of chemotherapy (standard therapy) pose major obstacles in cancer treatment, with multidrug resistance being a driving factor in chemotherapy failure. The past three decades have been the stage for intense research activity on the topic of nanomedicine, which has resulted in many nanotherapeutics with reduced toxicity, increased bioavailability, and improved pharmacokinetics and therapeutic efficacy employing smart drug delivery systems (SDDSs). Polymeric micelles (PMs) have become an auspicious DDS for medicinal compounds, being used to encapsulate hydrophobic drugs that also exhibit substantial toxicity. Through preclinical animal testing, PMs improved pharmacokinetic profiles and increased efficacy, resulting in a higher safety profile for therapeutic drugs. This review focuses on PMs that are already in clinical trials, traveling the pathways from preclinical to clinical studies until introduction to the market.

## 1. Introduction

Cancer corresponds to a heterogeneous group of malignant diseases in which cells divide abnormally without control, with the potential to invade other tissues [1,2,3]. Cancer ranks second among the main causes of death worldwide (particularly in industrialized countries), and its incidence is expected to increase. According to the World Health Organization (WHO), global cancer statistics (2022) pointed to 20 million new cases and 9.7 million deaths, with a 77% increase expected by 2050 [4]. Lung, breast, colorectal, prostate, and stomach cancers are the most frequent ones (Table 1) as stated by the Global Cancer Observatory (GLOBOCAN) of the International Agency for Research on Cancer (IARC).

Cancer is a multifactorial disease that can be triggered by genetic predispositions, environmental influences, and lifestyle choices. Some risks are already known, including immune system diseases, high-fat diets, tobacco use, excessive alcohol consumption, and viral infections [5]. The incidence is influenced by genetic conditions, age, ethnicity, and geography. The majority of neoplasms are sporadic, however, genetic inheritance is known to play a significant role [5].

### 1.1. The Tumor Microenvironment

Cancer complexity stems from both inter- and intratumoral heterogeneity and dynamic cell plasticity, which determines cancer cell progression, spread, and treatment resistance [2]. A network of signal transduction pathways, particularly those that promote the epithelial-to-mesenchymal transition and metabolic remodeling, are involved in the transformational nature of cancer and influence the evolutionary trajectory of cancer cells [2,6].

**Table 1:** Global prevalence, incidence, and years lived with disability (YLDs) ascribed to cancer for males, females, and both genders in 2021 with percentage change (numbers in parentheses) between 2010 and 2021. Data from Global Burden of Disease Collaborative Network 2022 [7].

Cancer Type	Prevalence Cases (Millions)	Incidence Cases (Millions)	YLDs Counts (Millions)
Breast	20.6 (34.0%)	2.12 (32.8%)	1.48 (31.8%)
female	20.3 (33.8%)	2.08 (32.6%)	1.45 (31.5%)
male	0.320 (53.6%)	0.0388 (46.7%)	0.0293 (49.0%)
both	20.6 (34.0%)	2.12 (32.8%)	1.48 (31.8%)
Colorectal			
female	4.92 (35.1%)	0.931 (31.4%)	0.450 (32.6%)
male	6.76 (44.2%)	1.26 (39.4%)	0.632 (41.4%)
both	11.7 (40.2%)	25.6 (35.9%)	1.08 (37.6%)
Prostate			
male	10.4 (33.5%)	1.32 (31.2%)	0.848 (30.4%)
Trachea, bronchus, and lung			
female	1.15 (37.0%)	0.779 (37.3%)	0.188 (35.8)
male	2.10 (24.0%)	1.50 (21.2%)	0.361 (21.4%)
both	3.25 (28.3%)	2.28 (26.2%)	0.548 (26.0%)
Stomach			
female	0.695 (9.8)	0.397 (9.8%)	0.101 (9.8%)
male	1.70 (14.2%)	0.833 (9.5%)	0.224 (1.0%)
both	2.39 (12.9%)	1.23 (9.6%)	0.326 (10.6%)
Liver			
female	0.219 (21.4%)	0.165 (28.2%)	0.0391 (26.8%)
male	0.521 (23.3%)	0.364 (25.0%)	0.0874 (25.1%)
both	0.739 (22.7%)	0.529 (26.0%)	0.127 (25.6%)
Pancreatic			
female	0.201 (36.7%)	0.235 (38.5%)	0.0475 (37.6%)
male	0.238 (35.9%)	0.274 (38.1%)	0.0561 (36.8%)
both	0.439 (36.3%)	0.509 (38.3%)	0.104 (37.2%)

Despite tumor heterogeneity, the tumor microenvironment (TME) shares common features that contribute to cancer development [8,9]. Various cell types, including mesenchymal stromal cells (MSCs), tumor endothelial cells (TECs), pericytes, and infiltrating immune cells (lymphocytes, neutrophils, tumor-associated macrophages (TAMs), and mast cells) are found in the TME, which also includes cancer cells, cancer-associated fibroblasts (CAFs), and the extracellular matrix (ECM) [8,9].

Genetic alterations in tumor cells lead to hyperplasia, uncontrolled growth and proliferation, and resistance to apoptosis. Several proteins, enzymes, cell surface receptors, growth factors, inflammatory mediators (e.g., cytokines and chemokines), and antigens are overexpressed in tumor cells [10,11]. The increased expression of matrix metalloproteinases (MMPs), responsible for the degradation of ECM proteins, plays an important role in TME remodeling associated with cell proliferation, migration, angiogenesis, and metastasis. Additionally, cancer cells often overexpress integrins, which are cell-adhesion transmembrane receptors involved in angiogenesis, recruitment of inflammatory cells, and tumor invasiveness [10,11]. Rapid proliferation of cancer cells is also associated with an increased demand for folate and iron. Thus, folate receptors (FRs) and transferrin receptors (TfRs), which mediate cellular uptake of folate and iron, respectively, are often overexpressed in cancer cells [10,11].

Moreover, the high oxygen requirement of fast proliferating tumor cells and the compromised tumor vasculature lead to low oxygen supply, and the resulting hypoxia environment triggers a metabolic shift from mitochondrial oxidative phosphorylation (the main pathway for energy production in normal cells) towards aerobic glycolysis [8,9]. Known as the Warburg effect [12], aerobic glycolysis leads to preferential glucose uptake and conversion into lactic acid, resulting in the acidic extracellular pH characteristic of solid tumors. This metabolic shift is mediated by hypoxia-inducible factor-1 (HIF-1), a transcription factor upregulated in cancer cells that responds to hypoxia by activating genes involved in glycolysis, angiogenesis, and cell survival [8,9]. Tumor hypoxia enhances the production of reactive oxygen species (ROS) and oxidative stress and affects the redox status of the TME by upregulating antioxidant enzymes involved in the synthesis of glutathione (GSH), an important intracellular antioxidant that regulates the cellular redox state by scavenging reactive oxygen and nitrogen species [8,9]. Despite the redox potential difference between the intra- and extracellular milieu of normal cells, this differential is increased in cancer cells due to 2- to 4-fold higher GSH content in the cytosol and subcellular organelles, contributing to the reductive TME.

Additionally, hypoxia drives the release of vascular endothelial growth factor-A (VEGF-A) that binds the VEGF receptor 2 (VEGFR2) at the surface of neighboring endothelial cells, inducing angiogenesis [8,9]. The overexpression of VEGF-A at the TME enhances neo-angiogenesis, producing primitive vasculature networks characterized by dysfunctional blood vessels with irregular and leaky lumen, which contribute to increased interstitial fluid pressure. VEGF-induced primitive vasculatures are shared in various solid tumors, promoting tumor growth, invasion, and metastasis [9]. Furthermore, TECs are known to overexpress α_v_β_3_, an integrin involved in the regulation of the sprouting ability of endothelial cells during angiogenesis [9].

**Figure 1:**
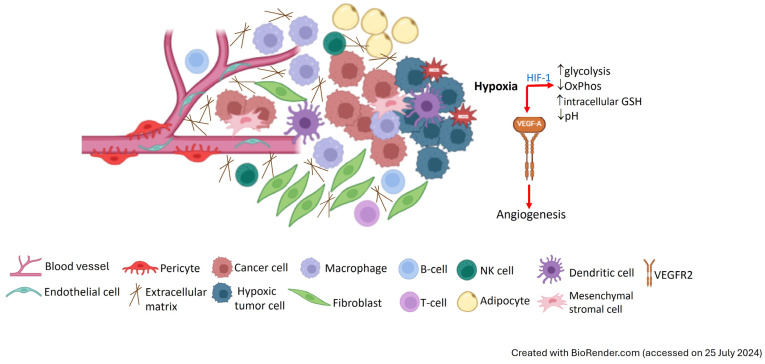
Characteristic features of the tumor microenvironment.

The hallmarks of the TME are summarized in Figure 1.

### 1.2. Cancer Chemotherapy

Chemotherapy is an effective treatment strategy for cancer, often combined with surgery and/or radiotherapy, depending on the tumor stage [13,14]. Despite the development of novel cancer treatment options, such as small-molecule targeted anticancer drugs (e.g., oncogene-targeted tyrosine kinase inhibitors, TKIs), immunotherapies (e.g., immune checkpoint inhibitors), and gene therapies (e.g., plasmid DNA, small interfering RNA, and microRNAs), conventional chemotherapy relying on cytotoxic drugs to kill and/or inhibit the growth and proliferation of cancer cells remains the first-line treatment for many cancers [1,13]. 

Traditional chemotherapeutic drugs are classified according to their primary mechanism of action (Table 2), which typically involves interaction with DNA and disruption of the cell cycle, although other secondary modes of action, such as the production of ROS and interference with mitochondrial pathways, also contribute to their cytotoxicity, ultimately leading to cell death in tumor tissues [13].

Chemotherapy is associated with toxic side effects due to the broad spectrum of activity and narrow therapeutic window of cytotoxic drugs, which do not distinguish between cancer cells and normal (healthy) cells. Most chemotherapeutic agents preferentially attack rapidly multiplying cells, such as cancer cells, but also bone marrow, gastrointestinal tract, and hair follicle cells [13]. Common adverse events (AEs) associated with cytotoxic anticancer drugs include myelosuppression, neutropenia, neurotoxicity, nephrotoxicity, hepatotoxicity, mucositis, nausea, vomiting, diarrhea, alopecia, cutaneous reactions, anemia, body weight loss, fatigue, and an increased risk of infections due to immunosuppression [1,13,15].

**Figure 2:**
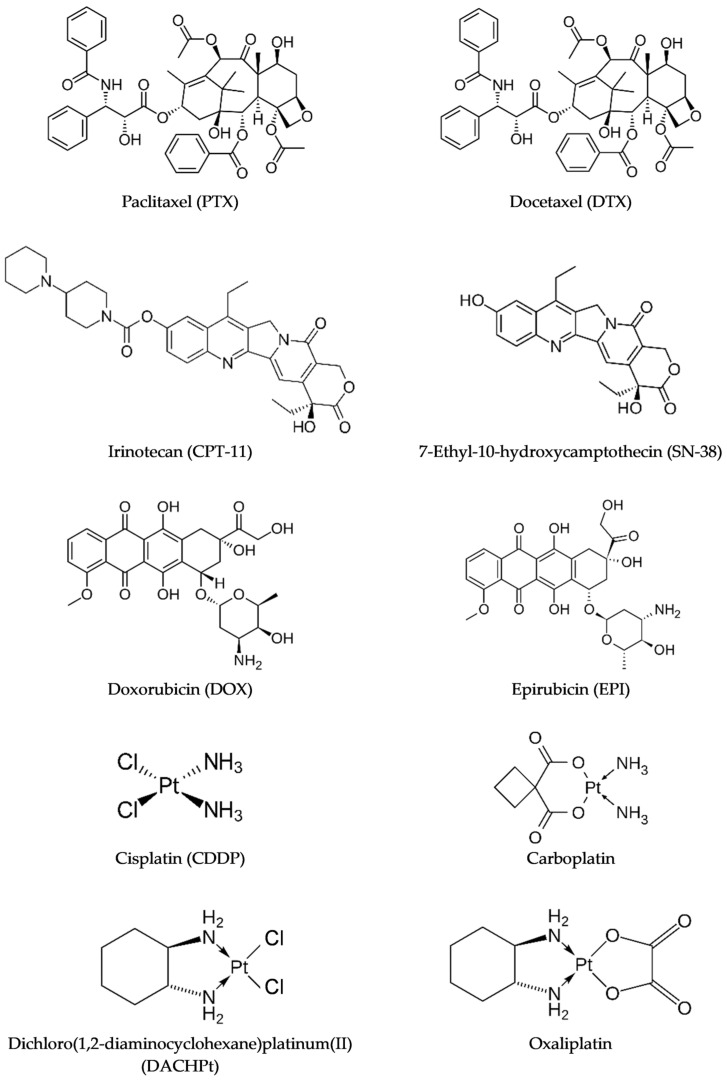
Some chemotherapeutic drugs under development as polymeric micellar formulations currently in different phases of clinical trials.

Moreover, most cytotoxic drugs in the clinic are highly hydrophobic (Figure 2), suffering from poor water solubility and low bioavailability, being administered as intravenous (IV) infusions (using body surface area dosing) at repeated, regular intervals (treatment cycles) to allow the recovery of normal tissues [13,15]. Nevertheless, neo-angiogenesis, dysfunctional tumor vasculature, increased interstitial fluid pressure, and efflux pumps, namely P-glycoprotein (P-gp), hinder intracellular therapeutic concentrations of cytotoxic drugs reaching the tumor site. The subtherapeutic drug availability in cancer cells can lead to the development of multidrug resistance (MDR), therefore a higher dose, often the maximum tolerated dose (MTD), is usually applied, causing systemic toxicity and potential severe AEs [13,15].

Combination chemotherapy involving the “cocktail” administration of multiple chemotherapy drugs simultaneously, preferentially with different mechanisms of action and non-overlapping toxicities, has shown considerable promise in overcoming MDR, preventing disease recurrence, and extending the survival of cancer patients when compared to monotherapy regimens [13,16]. Similarly, the combination of chemotherapy with phototherapy, gene therapy, and immunotherapy produces synergistic antitumoral effects and allows lower doses of the cytotoxic agent, reducing toxic side effects and enhancing the sensitivity of cancer cells toward the chemotherapeutic drug [16,17,18]. 

Improving the selectivity of anticancer drug delivery towards cancer cells while sparing normal (healthy) cells and tissues is a major challenge in cancer chemotherapy considering tumor heterogeneity and complexity. Therefore, targeting the TME or its (a)cellular components, which can reprogram tumor initiation, growth, invasion, metastasis, and therapy response, is a promising therapeutic strategy and nanomedicines are particularly suited to the task [19]. Several nanoformulations of hydrophobic cytotoxic drugs have been approved by the United States Food and Drug Administration (FDA) for cancer chemotherapy and are currently in the market, including Doxil^®^/Lipodox^®^ (liposomal formulation of doxorubicin) and Abraxane^®^ (nanoparticle albumin-bound paclitaxel), while many others are in clinical trials, including polymeric micelles [20]. The role of nanotechnology in the development of effective anticancer therapies for targeted and controlled drug delivery at the tumor site, thus improving drug efficacy and reducing systemic toxicity, is discussed in the next section.

## 2. The Role of Nanotechnology in Cancer Chemotherapy

The nanotechnological approach, involving drug encapsulation in polymer- or lipid-based nanoparticles (NPs), is a promising strategy for the delivery of hydrophobic chemotherapeutic agents. The nanocarriers solubilize the drug and protect it from chemical and enzymatic degradation, prolonging circulation in the bloodstream while simultaneously avoiding systemic toxicity [1]. Drugs can be physically encapsulated or chemically conjugated to the NP via labile bonds (prodrugs). In the former case, drug release rate is controlled by erosion of the biodegradable core material, diffusion across the polymeric matrix core, or polymer swelling followed by drug diffusion, while in the latter, drug release requires activation via bond cleavage by small molecules or enzymes [21,22]. 

Size is crucial for cellular uptake, drug release kinetics, biodistribution, and toxicity of NPs. NPs are usually internalized through endocytic pathways, becoming trapped in lysosomes and endosomes [1,23]. The cellular uptake of NPs with sizes in the range 20–100 nm involves caveolin-mediated endocytosis while larger NPs, within the submicron range (100–350 nm), are mainly internalized through clathrin-mediated endocytosis [23]. Therefore, targeting drug-loaded NPs to specific cellular organelles, such as endosomes and lysosomes, can circumvent recognition by drug efflux pumps, like P-gp, through internalization by endocytosis, thus overcoming MDR in cancer cells. Other mechanisms, like phagocytosis and micropinocytosis, may also contribute to NP internalization [23]. Besides size, cellular uptake is also influenced by surface charge of the NP. Cell membranes are negatively charged, which enhances cellular uptake of NPs with net positive surface charge (positive zeta potential) over negatively charged ones due to attractive electrostatic interactions, while selective uptake of anionic NPs by phagocytic cells has been reported [1,23,24]. 

NPs interact with serum proteins, which adsorb at the surface of the nanocarrier forming a protein corona that prevents NP agglomeration and reduces their toxicity, but also enhances their recognition and clearance by the mononuclear phagocytic system (MPS), which limits NP delivery and distribution [1,22,23,25]. Surface functionalization of NPs can reduce their cytotoxicity and promote cellular uptake by inhibiting protein corona formation in the presence of serum proteins. Hydrophilic surface coating minimizes protein adsorption and prolongs circulation in the bloodstream by escaping the MPS in the liver and spleen [1,22,23]. Poly(ethylene glycol) (PEG) is a hydrophilic, non-ionic synthetic polymer that is FDA-approved for clinical use and most often selected as hydrophilic coating due to its good biocompatibility and non-immunogenicity [1,23,26]. PEG is water-soluble and in aqueous environments forms a highly hydrated, dense brush-like shell that ensures NP solubility and stability, hindering NP aggregation as well as protein binding and opsonization, thus prolonging the NP circulation time upon systemic administration. Furthermore, PEG offers the additional advantage of being easily functionalized with appropriate ligands for targeted drug delivery, contributing to improving the efficacy and safety of anticancer drugs [26]. However, PEG is not biodegradable, and its excretion is dependent on the molecular weight (MW) of the polymer. Low-MW PEGs mainly undergo renal clearance by passive glomerular filtration while those with high MW are predominantly excreted into bile [27]. The MW threshold for kidney clearance of PEGs has been determined as 30 kDa, and PEGs with MWs in the range 2–15 kDa are often chosen to allow for complete renal excretion of the polymer [27]. 

Moreover, NPs can effectively target the drug to the local site(s) of action, i.e., tumor tissues, which can be achieved by passive or active targeting. 

### 2.1. Tumor-Targeting Mechanisms

#### 2.1.1. Passive Targeting

In passive targeting, NPs accumulate in tumor tissues due to the compromised leaky vasculature, which allows extravasation of nanosized particles (10–100 nm) that become trapped in the TME because of poor lymphatic drainage, a phenomenon known as the enhanced permeability and retention (EPR) effect [21,22,25]. After accumulating in tumors, NPs can act as intracellular Trojan horses, selectively delivering the chemotherapeutic drugs to their subcellular targets, thus overcoming drug resistance mechanisms. EPR-based chemotherapeutics are known to alter the drug pharmacokinetics and biodistribution, minimizing the plasma concentration peak (*C*_max_) and increasing the area under the concentration–time curve (AUC), both in plasma and in tumor, thus providing longer exposure to therapeutic levels of the drug at the target site that contribute to improving drug efficacy and safety [21,22].

#### 2.1.2. Active Targeting

Active targeting makes use of the overexpression of certain cell surface receptors and antigens in cancer cells, aiming at increasing NP accumulation in tumor tissues and simultaneously enhancing selective uptake via receptor-mediated endocytosis [10,11,28]. Functionalization of the surface of NPs by conjugation with appropriate targeting ligands, such as folate, transferrin, monoclonal antibodies (mAbs), peptides, carbohydrates, and aptamers, is an efficient strategy for specific and selective drug delivery to cancer cells or intracellular components [10,11,22,28]. 

The targeting ligands can identify a variety of representative tumor biomarkers, such as FR, TfR, insulin receptor (IR), estrogen receptor α (ERα), prostate-specific membrane antigen (PSMA), mucin-1 (MUC1), nucleolin, and human epidermal growth factor receptor 2 (HER2) [10,11,29,30,31]. TfR is overexpressed in cancer cells, and its endothelium expression is restricted to the endothelial cells forming the blood–brain barrier (BBB), which allows drug targeting to the central nervous system (CNS). Transferrin-targeted NPs can cross the BBB and enhance cellular uptake and brain accumulation of antiglioma drugs with poor BBB permeability [10,11,28]. NPs functionalized with carbohydrate moieties (e.g., galactose, lactose, glucose, or mannose) target the asialoglycoprotein receptor (ASGPR) overexpressed in hepatocellular carcinoma cells for selective drug delivery with high affinity [10,28]. Among the peptide ligands, arginine–glycine–aspartic acid (RGD), or other peptides containing this amino acid sequence, are often used for targeting α_v_β_3_ integrin receptors overexpressed in cancer cells and angiogenic endothelial cells in the tumor vasculature [10,11,28]. 

Antibody-targeted NPs, prepared by attaching mAbs or antibody fragments to the hydrophilic surface of the NP (usually PEG), provide broad diversity of targets and specificity of interaction [10,28]. Aptamers are synthetic single-stranded RNA or DNA oligonucleotides designed to bind specific molecular targets. Aptamers are smaller and less immunogenic than mAbs, show better tissue penetration, and resist enzymatic degradation in vivo (unlike peptides), being easily synthesized and modified [10,28,31]. 

### 2.2. Stimuli-Responsive Nanocarriers

Controlled drug release at the target site is crucial to achieve therapeutic concentrations. Stimuli-responsive “smart” NPs are designed for effective on-site drug release by undergoing changes in chemical structure or physicochemical properties in response to specific environmental stimuli, either endogenous or exogenous, or a combination of two or more stimuli, to improve targetability and combinatorial drug delivery [21,22,28,32,33].

#### 2.2.1. Endogenous Stimuli

Endogenous stimuli include pH, redox status, hypoxia, and upregulated enzymes characteristic of the TME. 

The acidic extracellular pH (6.5–6.9) in tumors due to the Warburg effect can be used to trigger drug release from pH-sensitive NPs, constructed by the introduction of ionizable chemical groups (“titratable” groups, such as amines or carboxylic acids) in the NP structure or by drug–NP conjugation through acid-labile linkers (e.g., ester, hydrazone), which are stable at physiological pH (7.4) but release their cargo in the acidic TME, induced by extensive protonation of the titratable group or hydrolysis of the linker, respectively [21,22,28,32,33,34,35]. The same strategy can be used for subcellular drug targeting to acidic organelles, namely endosomes (pH 6.5–6.9) and lysosomes (pH 4.0–5.0), ensuring effective intracellular drug concentrations [28,33]. 

By incorporating enzyme-labile linkages in the NP structure or in the conjugated drug, NPs can be engineered to release the encapsulated drug on demand, using enzymes overexpressed in the extracellular TME [21,22,24,32,36]. Proteinase substrates are commonly used for fabricating NPs with enzyme-responsive linkers [21,22,26,28,32,33]. For instance, NPs modified with short peptide substrates containing MMP-cleavable sequences release their cargo after exposure to MMP-2 overexpressed at the tumor site [26,28,33].

The difference in redox potential between the oxidative extracellular space and the reductive intracellular space, much richer in GSH, has been explored in the design of redox-responsive NPs. Redox-sensitive NPs containing disulfide bonds are stable in the bloodstream as well as in endocytic vesicles but release their cargo in the reductive TME upon reduction of the disulfide linker by cytosolic GSH, which is overexpressed in cancer cells [21,22,26,28,32,33]. ROS-responsive NPs able to respond to the altered oxidative microenvironment of tumor cells due to excessive ROS production have also been developed, mainly using thioether-based oxidation-sensitive polymers that exhibit variation in solubility in response to ROS overproduction and ROS-induced degradation [26,28,32,37,38,39].

#### 2.2.2. Exogenous Stimuli

Exogenous stimuli, such as temperature, magnetic field, light, ultrasound waves, and electrical fields, have also been used to trigger drug release from NPs accumulated at the target sites. Thermoresponsive NPs made from thermosensitive materials that exhibit a lower critical solution temperature (LCST), such as poly(*N*-isopropylacrylamide) (PNIPAM), release their cargo due to phase separation at temperatures above the LCST [21,22,28,32,33,40]. In drug targeting of thermoresponsive NPs to cancer cells, hyperthermia can be used as trigger. Magnetic NPs, such as iron oxide NPs, including Fe_3_O_4_ (magnetite), γ-Fe_2_O_3_ (maghemite), and α-Fe_2_O_3_ (hematite) as well as superparamagnetic iron oxide nanoparticles (SPIONs), release their cargo when placed under an oscillating magnetic field. The latter do not retain magnetization upon its removal, which avoids NP aggregation in the absence of the magnetic field [28,32,33,41]. The rise in temperature (40–44 °C) by the electromagnetic waves (magnetic hyperthermia) can also be used to enhance drug efficacy [42].

Light-sensitive NPs are promising drug delivery systems (DDSs) for spatiotemporally controlled release of drugs at target sites upon stimulation with ultraviolet (UV), visible, or near-infrared (NIR) light, depending on the chromophore incorporated in the NP [21,22,32,43]. Photoactive agents are already used in phototherapy and optical imaging in clinical practice, including fluorescence imaging and fluorescence-guided surgery [44]. Ultrasound-responsive NPs release their payloads under the influence of ultrasound waves, which penetrate deeper into the body than light, and can induce drug release by both thermal (hyperthermia) and mechanical effects (cavitating bubbles) [28,32,33,45,46]. Non-thermal effects associated with oscillating or cavitating bubbles can disrupt the nanocarrier and contribute to micropore formation in target cell membranes, enhancing membrane permeability and passive diffusion with subsequent intracellular drug accumulation [28,32,33].

Drug release from electric-field-responsive NPs is triggered by an applied electric field, which is easy to generate and control [21,22]. This type of NP can be constructed using conductive polymers, such as poly(pyrrole) (PPy), and their properties depend on dopant selection and MW of the drug [47]. Biotin (an essential vitamin involved in cellular carbohydrate, amino acid, and lipid metabolism) is often the dopant of choice, and the biotin-doped polymer is reacted with streptavidin (with four biotin-binding sites) with subsequent incorporation of a biotinylated drug. Electrical stimulation leads to the reduction of the PPy backbone and triggers the release of the biotin and the attached payload [47]. 

Among the numerous nanoparticulate drug delivery systems available, including nanoliposomes, polymeric NPs, polymeric micelles, dendrimers, polymersomes, lipid NPs, and inorganic NPs, polymeric micelles have emerged as promising platforms for targeted and controlled delivery of anticancer agents, as discussed in the next section.

## 3. Structure and Preparation of Polymeric Micelles

**Figure 3:**
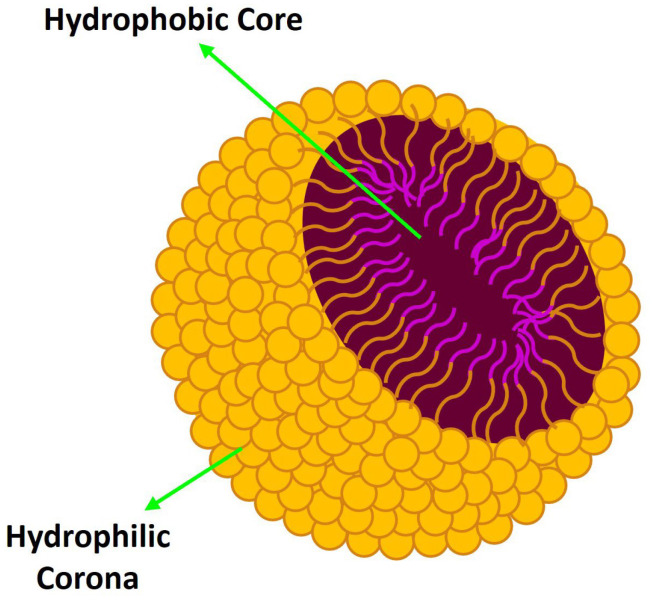
Schematic representation of a polymeric micelle.

Polymeric micelles (PMs) are spherical-shaped nanostructures with sizes typically ranging from 10 to 100 nm, formed by spontaneous self-assembly of amphiphilic block copolymers in an aqueous environment when the concentration reaches a critical value, known as the critical micelle concentration (CMC) [21,22]. PMs have a unique core–shell structure with the hydrophobic blocks forming an inner core surrounded by a shell of hydrophilic blocks creating a protecting layer (corona) at the micelle–water interface (Figure 3). The hydrophobic effect is the main driving force for the self-assembly process, which lowers the Gibbs energy of the system by removing the hydrophobic blocks from the aqueous milieu [21,22].

The size and stability (CMC) of PMs depend on the hydrophilic–lipophilic balance (HLB) of the block copolymers and their MW [21,22]. The properties of the hydrophobic block strongly influence drug-loading capacity, stability, and drug release behavior while the properties of the hydrophilic block modulate the in vivo pharmacokinetic profile [1,21,22]. The failure of NPs in clinical trials is often due to poor pharmacokinetic profile. Pharmacokinetic parameters for some PMs that have completed or are still in clinical trials are presented in Table 3.

### 3.1. Polymers Used for the Manufacturing of Polymeric Micelles

Both natural and synthetic polymers can be used in the construction of PMs. Despite their good biocompatibility, biodegradability, and low immunogenicity, natural biopolymers like chitosan, alginate, and hyaluronan are susceptible to chemical and enzymatic degradation, have an associated risk of microbial contamination, may trigger allergic reactions in susceptible individuals, and display limited batch-to-batch reproducibility [21,22]. On the other hand, synthetic polymers have predictable and controllable physicochemical properties, which can be tailored by appropriate choice of monomer units, type of polymerization reaction, formation of copolymers, as well as easy functionalization [21,22].

Biocompatibility and biodegradability are key criteria for selection of hydrophobic core-forming blocks. Poly(esters), like poly(D,L-lactic acid) (PDLLA), poly(lactic-co-glycolic acid) (PLGA), and poly(ε-caprolactone) (PCL), and poly(amino acids), like poly(L-aspartic acid) (pAsp) and poly(L-glutamic acid) (pGlu), are usually employed since they undergo in vivo hydrolysis of their ester and amide bonds to yield the respective monomers, carboxylic acids and L-amino acids, which can be eliminated by natural metabolic pathways [21,22]. The poly(esters), namely FDA-approved PLGA and PDLLA, are usually selected due to their good safety profile, since their biodegradation generates glycolic and/or lactic acids, which are ultimately degraded to carbon dioxide and water via the Krebs cycle [21,22]. The side chains in the residues of poly(amino acids) allow functionalization to further enhance hydrophobicity and loading ability or for covalent coupling of the drug, leading to self-assembled block copolymer–drug conjugate micelles [21,22].

Non-biodegradable poly(ethers), like poly(propylene oxide) (PPO), can also be used, namely in poloxamers (Pluronics^®^), which are amphiphilic triblock copolymers composed of hydrophilic poly(ethylene oxide) (PEO) and hydrophobic PPO disposed in a PEO–PPO–PEO arrangement [21,22]. These non-ionic copolymers are often used due to their biocompatibility, intrinsic stealth effect, and commercial availability. 

Among the available hydrophilic polymers, which include PEG, poly(vinyl alcohol) (PVA), poly(vinylpyrrolidone) (PVP), poly(ethylene imine) (PEI), poly(2-methyl-2-oxazoline) (PMeOx), poly(L-lysine) (PLL), and poly(2-hydroxyethyl methacrylate) (pHEMA), PEG is usually the polymer of choice due to its good biocompatibility, favorable safety profile, and ease of surface functionalization with appropriate ligands for targeted delivery to cancer cells or specific organelles [1,22,48,49].

The use of stimuli-responsive polymers leads to “smart” PMs, which usually release their cargo through micelle disassembly at the target site upon an endogenous or exogenous stimulus. Poly(aminoethyl acrylamide) (PAEA) and poly(2-(dimethylamino)ethyl methacrylate) (PDMAEMA) are pH-sensitive hydrophilic polymers while PNIPAM is also a thermosensitive polymer [21,22,24]. PNIPAM exhibits an LCST at 32 °C in aqueous media, corresponding to a reversible phase transition from an expanded coil state (hydrophilic) below the LCST to a collapsed globule state (hydrophobic) above the LCST through cooperative breaking of hydrogen bonding between the amide group of the polymer and water [21,22].

### 3.2. Crosslinked Polymeric Micelles

Although amphiphilic copolymers exhibit very low CMC (10^−6^–10^−7^ mol/L) when compared with low MW surfactants, extensive dilution after IV injection can lead to micelle dissociation and drug leakage with potential systemic toxicity. Thus, micelles can be crosslinked, either at the core or at the shell, to improve stability in the bloodstream and control drug delivery at the target site [29,50]. Despite shell-crosslinking imparting stabilization to PMs, it can also affect their hydrophilicity and stealth properties, therefore core-crosslinking is preferable for micelle stabilization [29,50]. Additionally, hydrophobic drugs physically encapsulated in the core-crosslinked polymeric micelles (CCPMs) can be attached to the crosslinkers of the core.

Covalent core-crosslinking of PMs is performed after micelle formation and usually requires side-chain- or end-group-functionalized block copolymers, employing radical polymerization or addition of a bifunctional agent as a crosslinker for PMs containing polymerizable groups (e.g., methacrylate) or reactive groups (e.g., carboxylic, amine), respectively, within the core [29,50]. Alternatively, core-crosslinking can be achieved under oxidative conditions through disulfide bridge formation if the PMs contain thiol groups, and the resulting crosslinked micelles are stable in the bloodstream, which contains very low levels of GSH (2–10 μmol/L compared with 0.5–10 mmol/L in the cytosol) [29,50]. These redox-responsive biodegradable micelles are cleaved and release their cargo in the reductive intracellular microenvironment of cancer cells overexpressing GSH. 

**Table 3:** Pharmacokinetic parameters for polymeric micellar formulations of anticancer drugs in clinical trials in comparison with other nanoformulations and conventional chemotherapeutic drugs in the market, obtained upon intravenous administration.

Formulation	Polymer	Drug	Drug-Loading Method	Size (nm)	Dose (mg/m^2^)	n	*C*_max_ (μg/mL)	*T*_max_ (h)	AUC_0→∞_ (μg·h/mL)	*t*_1/2z_ (h)	Ref.
Genexol^®^-PM	mPEG_2k_-PDLLA_1.75k_	PTX	Physical	25	390 ^a^	2	6.567 ± 1.120		27.490 ± 8.297	17.9 ± 1.0 (β)	[51]
					300 ^b^	3	3.107 ± 1.476		11.580 ± 4.277	11.4 ± 2.4 (β)	[51]
					180 ^a,b^	3	4.6758		7.4702	7.8	[52]
Abraxane^®^	Albumin NP	PTX	Nab	130	300 ^a,b^	5	13.520		17.610	14.6	[53]
Taxol^®^	Cremophor^®^ EL (surfactant)	PTX	Free drug		250 ^a^	5	19.167 ± 5.324		35.018 ± 12.758	0.33 ± 0.14 (α) 3.10 ± 1.08 (β)	[54]
					175 ^b^	3	8.641 ± 0.985		14.523 ± 1.936	0.30 ± 0.10 (α) 3.27 ± 0.95 (β)	[54]
PM-PTX	mPEG-PDLLA	PTX	Physical	20	390 ^a^	3	4.044 ± 1.226		19.381 ± 5.025	19.3 ± 3.29	[55]
					300 ^b^	6	3.736 ± 1.220		13.751 ± 3.440	16.6 ± 6.55	[55]
Nanoxel^®^	PVP-PNIPAM	PTX	Physical	80–100	375 ^a^	6			32.758		[56]
					300 ^b^	6			23.632		[56]
NK105	PEG-p(Asp)	PTX	Physical	85	180 ^a^	4	45.6278 ± 8.6430		454.5 ± 119.1	11.3 ± 0.6	[57]
					150 ^b^	7	40.1699 ± 5.5334		369.8 ± 35.2	10.6 ± 1.3	[57]
					100 ^a^	6	27.6 ± 5.3	0.80 ± 0.30	390 ± 64	12.3 ± 1.3	[58]
					80 ^b^	3	22.0 ± 3.3	0.80 ± 0.30	302 ± 104	12.7 ± 0.8	[58]
CPC634 (CriPec^®^ DTX)	mPEG_5k_-pHPMA-Lac_n_	DTX	Conjugation	65	100	3	44.1161 ± 8.6453 (T) 0.3219 ± 0.1206 (Rel)	3.3 ± 2.3 (T) 1.8 ± 0.3 (Rel)	1836.280 ± 385.084 (T) 8.4244 ± 0.5625 (Rel)	35.0 ± 3.2 (T) 44.9 ± 9.9 (Rel)	[59]
					60 ^b^	5	27.1444 ± 7.9993 (T) 0.2173 ± 0.0919 (Rel)	1.5 ± 0.4 (T) 1.4 ± 0.4 (Rel)	973.987 ± 246.491 (T) 4.0675 ± 2.9740 (Rel)	31.6 ± 1.3 (T) 39.7 ± 9.4 (Rel)	[59]
					75	26	44.4 (T) 0.262 (Rel)		1530 (T) 7.41 (Rel)	38.03 (T) 59.05 (Rel)	[60]
Conventional DTX	Tween^®^ 80 (surfactant)	DTX	Free drug		75	26	2.88		5.74	100.09	[60]
Taxotere^®^	Tween^®^ 80 (surfactant)	DTX	Free drug		100	4	2.41 ± 0.35		5.93 ± 0.53	18.5 ± 10.7	[61]
NK012	PEG_12k_-p(Glu)_7k_	SN-38	Conjugation	20	28	9	19.1 ± 3.9 (PB) 0.114 ± 0.031 (Rel) 0.04 ± 0.01 (G)	0.7 ± 0.3 (PB) 0.8 ± 0.3 (Rel) 6 ± 0 (G)	294 ± 62 (PB) 2.12 ± 0.83 (Rel) 2.28 ± 0.57 (G)	137 ± 19 (PB) 209 ± 25 (Rel) 205 ± 20 (G)	[62]
					28	6	20.0 ± 4.6 (PB) 0.138 ± 0.024 (Rel) 0.0721 ± 0.0241 (G)	0.75 ± 0.27 (PB) 0.56 ± 0.23 (Rel) 6.21 ± 0.51 (G)	287 ± 60 (PB) 2.13 ± 0.28 (Rel) 4.95 ± 1.78 (G)	153 ± 27 (PB) 282 ± 101 (Rel) 382 ± 179 (G)	[63]
CPT-11		CPT-11	Free drug		250	5	7.58 ± 3.06		27.86 ± 4.46	4.5	[63]
SN-38		SN-38	CPT-11 active metabolite		250	5	0.072 ± 0.018		0.876 ± 0.301	13.9	[63]
NK911	PEG_5k_-p(Asp)_4k_	DOX	Conjugation and physical	40	67 ^a^	6			4.1741 ± 0.4712	0.13 ± 0.02 (α) 2.9 ± 0.5 (β) 73.6 ± 21.4 (γ)	[64]
					50 ^b^	11			3.2627 ± 0.4252	0.12 ± 0.01 (α) 2.8 ± 0.3 (β) 64.2 ± 8.9 (γ)	[64]
Doxil^®^	HSPC:Chol: mPEG_2k_-DSPE liposomes	DOX	Remote, via transmembrane ammonium sulfate gradient	<100	50	14			902 (T) 893 (LB)	1.4 (α) (T) 45.9 (β) (T) 2.3 (α) (LB) 46.2 (β) (LB)	[65]
Adriamycin^®^	(DOX hydrochloride)	DOX	Free drug		50	4			3.5	0.06 (α) 10.4 (β)	[65]
SP1049C	Pluronics^®^	DOX	Physical	22–27	70 ^a,b^	7			1.62–4.20	0.08–0.13 (α) 1.07–3.05 (β) 21.3–56.5 (γ)	[66]
NC-6300	PEG_12k_-p(Asp)	EPR	Conjugation	40–80	185 ^a^	4	62.2 ± 10.0 (T) 0.357 ± 0.153 (Rel)	2.125 ± 3.92 (T) 6.1 ± 11.9 (Rel)	2835.3 ± 313.8 (T) 12.7 ± 7.1 (Rel)	25.0 ± 1.11 (T) 32.1 ± 5.1 (Rel)	[67]
					150 ^b^	4	49.4 ± 15.1 (T) 0.459 ± 0.354 (Rel)	0.167 ± 0.00 (T) 14.1 ± 22.9 (Rel)	2126.5 ± 573.0 (T) 17.1 ± 13.2 (Rel)	23.6 ± 1.09 (T) 26.3 ± 6.9 (Rel)	[67]
NC-6004	PEG-p(Glu)	CDDP	Metal complexation	30	135 ^a,b^		50.08 ± 9.84 (T)		2611.0 ± 520.7 (T)	91.2 ± 19.5 (T)	[68]
					120 ^a^	3	85.4 ± 10.8 (T) 84.6 ± 8.1 (mic) 0.131 (UF Pt)	4.4 ± 2.5 (T) 3.1 ± 1.5 (mic) 26.4 (UF Pt)	4377 ± 563 (T) 3857 ± 1171 (mic) 22.9 (UF Pt)	158 ± 48 (T) 87 ± 37 (mic) 115 (UF Pt)	[69]
					90 ^b^	6	60.8 ± 12.5 (T) 42.4 ± 20.3 (mic) 0.205 ± 0.114 (UF Pt)	5.2 ± 2.2 (T) 4.8 ± 2.4 (mic) 20.5 ± 7.6 (UF Pt)	2836 ± 554 (T) 1579 ± 939 (mic) 22.6 ± 10.0 (UF Pt)	129 ± 40 (T) 39 ± 27 (mic) 123 ± 44 (UF Pt)	[69]

^a^ MTD; ^b^ RD. Abbreviations: Asp, aspartate; AUC, area under the plasma concentration–time curve; CDDP, cisplatin; Chol, cholesterol; *C*_max_, maximum plasma concentration; CPT-11, irinotecan; DOX, doxorubicin; DSPE, distearoyl phosphatidylethanolamine; DTX, docetaxel; EPR, epirubicin; G, glucuronide; Glu, glutamate; HPMA, *N*-(2-hydroxypropyl)methacrylamide; HSPC, hydrogenated soy phosphatidylcholine; Lac, lactate; LB, liposome-bound; mic, micellar; MTD, maximum tolerated dose; Nab, nanoparticle albumin-bound; NP, nanoparticle; PB, polymer-bound; PDLLA, poly(D,L-lactic acid); PEG, poly(ethylene glycol); PNIPAM, poly(N-isopropylacrylamide); PTX, paclitaxel; PVP, poly(vinylpyrrolidone); RD, recommended dose; Rel, released; SN-38, 7-ethyl-10-hydroxycamptothecin; T, total; *t*_1/2z_, terminal half-life; *T*_max_, time to reach peak plasma concentration; UF Pt, ultrafiltrable platinum.

Non-covalent core-crosslinking has also been attempted, and functionalization of the hydrophobic copolymer with aromatic pendant groups (e.g., benzyl) increases micelle stability via crosslinked π–π-stacking interactions, simultaneously enhancing the loading of chemotherapeutic drugs containing aromatic rings, such as taxanes and anthracyclines (e.g., NK911 and NC-6300, Table 2) [50]. Moreover, since π–π stacking is a pH-sensitive force, these PMs constitute pH-responsive systems that release their payload in the acidic TME. For transition metal complexes, like the platinum-based chemotherapeutic agents, the drug itself can be used as a crosslinker, leading to core-crosslinked micelles through polymer–metal complexation and allowing for drug release upon ligand exchange at the target site (e.g., NC-6004 and NC-4016, Table 2) [50].

Core-crosslinking has been shown to improve in vivo stability, circulation time, and biodistribution of PMs, resulting in higher accumulation at the target site [29,50]. However, drug leakage may still occur, requiring drug attachment to the micellar core through covalent (and reversible) bonding to ensure drug retention during circulation and enable controlled and sustained release at the target site. The cocrosslinked prodrugs thus obtained show significantly improved biodistribution and pharmacokinetic profiles (e.g., CricPec^®^ Docetaxel, Table 2) [50].

## 4. Polymeric Micelles for Cancer Chemotherapy

**Figure 4:**
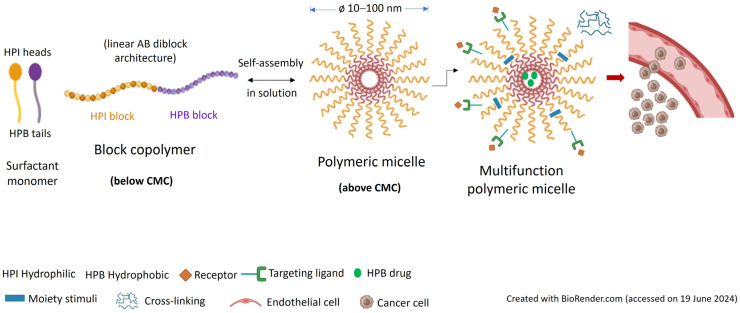
Multifunctional drug-loaded polymeric micelles for targeting cancer.

PMs have been extensively studied as smart DDSs for hydrophobic antitumor drugs for targeted cancer chemotherapy [21,22] (Figure 4). PMs loaded with two or more chemotherapeutic agents for targeted and controlled codelivery to cancer cells provide synergistic effects, hinder MDR, and avoid the side effects and multiple injections required in combination therapy regimens involving the free drugs [70,71,72]. Triolimus is a multidrug-loaded PM containing three complementary anticancer agents: paclitaxel, a microtubule stabilizer; rapamycin (sirolimus), the mammalian target of rapamycin (mTOR) inhibitor and potent immunosuppressor; and tanespimycin, a prototypical inhibitor of the 90 kDa heat shock protein (Hsp90) able to target compensatory pathways activated by mTOR inhibition [73]. The formulation exhibited potent synergistic cytotoxic activity in vitro against several human cancer cells lines [73] and in vivo in heterotopic and orthotopic tumor xenograft models [74]. Triolimus was granted orphan drug designation by the FDA for the treatment of angiosarcoma, in 2015 [75]. A similar formulation, obtained after replacement of paclitaxel by docetaxel, showed synergistic antitumor effects in a genetic mouse model of prostate cancer without inducing systemic toxicity [76]. 

Moreover, multifunctional PMs loaded with both imaging and chemotherapeutic agents, which allow simultaneous diagnosis and treatment, provide a promising theranostic platform for personalized cancer therapy that maximizes drug specificity and efficacy [28,77]. Several polymeric micellar formulations for the delivery of antitumor drugs or imaging agents are under different stages of clinical development while others have already been approved in several countries and are currently in the clinic. Information regarding the various polymeric micellar formulations in clinical trials is summarized in Table 4 and will be discussed in the following sections.

### 4.1. Taxane Micellar Formulations

Taxanes are natural diterpenoids isolated from yew with potent cytotoxic activity, being among the most clinically used chemotherapeutic drugs despite their narrow therapeutic window [78]. Paclitaxel (PTX), isolated from the bark of the Pacific yew tree (*Taxus brevifolia*), is widely used in the treatment of breast, ovarian, esophageal, bladder, prostate, and cervical cancers, non-small cell lung carcinoma (NSCLC), head and neck cancer, and melanoma and also as second-line treatment for Kaposi’s sarcoma [78]. PTX is a microtubule-stabilizing agent that binds to tubulin and inhibits microtubule disassembly, inducing mitotic arrest and cell apoptosis [78]. PTX is poorly water-soluble (0.3 μg/mL) [78,79], being conventionally formulated in a vehicle composed of a 50:50 (*v*/*v*) mixture of Cremophor^®^ EL (polyethoxylated castor oil) and ethanol (Taxol^®^, Bristol-Myers Squibb Co., Princeton, NJ, USA), which is diluted in normal saline or 5% dextrose solution for IV administration [78,79]. However, the Taxol^®^ formulation is commonly associated with several AEs, such as myelosuppression, neutropenia, anemia, nephrotoxicity, neurotoxicity (mainly peripheral neuropathy), and hypersensitivity reactions, the latter attributed to the high concentration of Cremophor^®^ EL (CrEL) surfactant required to solubilize the drug [78,80]. Premedication with corticosteroids (e.g., dexamethasone) and antihistamines, both H_1_ (e.g., diphenhydramine) and H_2_ receptor antagonists (e.g., cimetidine or ranitidine), is thus required to avoid the severe hypersensitivity reactions due to systemic exposure to high CrEL amount [78]. Additionally, since CrEL can leach plasticizers like di(2-ethylhexyl)phthalate (DEPH) from polyvinyl chloride (PVC) materials, Taxol^®^ administration requires PVC-free infusion systems (e.g., polyethylene-lined) with in-line filtration due to the risk of drug precipitation upon dilution [78,80]

Docetaxel (DTX) is a second-generation taxane obtained by semi-synthesis from 10-deacetyl baccatin III found in the needles of the European yew tree (*Taxus baccata*), being more potent than PTX at inhibiting microtubule depolymerization [81]. Like PTX, DTX is a microtubule-stabilizing agent approved in 2012 [82] and indicated as first-line chemotherapy for breast cancer, NSCLC, prostate cancer, head and neck squamous cell carcinoma (HNSCC), and stomach adenocarcinoma [83].

Due to poor water solubility (3 μg/mL) [81], DTX is solubilized using polysorbate 80 (Tween^®^ 80) and ethanol as cosolvent in the conventional commercial formulation, Taxotere^®^ (Sanofi-Aventis, Bridgewater, NJ, USA). The presence of the surfactant has been associated with AEs, including acute hypersensitivity and systemic immune reactions, hypotension, cutaneous reactions, fluid retention, and peripheral neuropathy [84]. Therefore, Taxotere^®^ administration requires premedication with oral corticosteroids [84].

#### 4.1.1. Genexol^®^-PM

Genexol^®^-PM, developed by Samyang Biopharmaceuticals Corporation (Seoul, South Korea), is a polymeric micellar formulation of PTX made from a low-MW, non-toxic, and biodegradable amphiphilic diblock copolymer composed of monomethoxy PEG (mPEG_2k_) and PDLLA_1.75k_ at a 60:40 weight ratio that self-assembles to form 25 nm NPs [79,85,86]. Genexol^®^-PM is free of CrEL, like Abraxane^®^, and a nanoparticle albumin-bound (nab)-paclitaxel formulation (ABI-007, Abraxis BioScience, Los Angeles, CA, USA) [79], which allows higher dose administration compared to Taxol^®^ without requiring premedication to prevent hypersensitivity reactions. Contrary to Abraxane^®^, Genexol^®^-PM does not contain human serum albumin, which is associated with a theoretical risk of viral transmission [75]). Moreover, Genexol^®^-PM, with a size around 25 nm, is able to penetrate tumor cells more easily and faster than nab-paclitaxel (Abraxane^®^), with a size around 130 nm, through the EPR effect.

Preclinical studies have shown that PTX biodistribution after administration of Genexol^®^-PM displayed 2- to 3-fold higher levels in several tissues, including liver, spleen, kidneys, lungs, heart, and tumor, as compared to Taxol^®^, with the highest PTX concentration found in the tumor [85]. The higher drug uptake and accumulation in tumor tissue are the result of polymeric micellar PTX targeting tumor cells through the EPR effect [85]. Despite showing comparable in vitro cytotoxicity against different human cancer cell lines, the in vivo antitumor efficacy of Genexol^®^-PM was significantly higher than that of Taxol^®^ [85]. Genexol^®^-PM is also more effective than Taxol^®^ as a radiosensitizer in chemoradiation therapy of NSCLC in the preclinical setting, preferentially accumulating in tumors and leading to lower PTX exposure of normal lung tissue than Taxol^®^ [86]. Furthermore, Genexol^®^-PM exhibited a controlled and sustained drug release profile, with 40% PTX release after 16 h and 95% after 48 h, which can increase the synergistic effects of chemotherapy and radiation therapy [86]. 

Unlike Taxol^®^, Genexol^®^-PM exhibits a linear pharmacokinetic profile, as observed in patients with advanced malignancies [51] and solid tumors [52]. The plasma AUC_0→∞_ and *C*_max_ of Genexol^®^-PM in cancer patients revealed lower values than equivalent doses of Taxol^®^ [51], since PTX entrapment in circulating CrEL micelles inhibits the partition of PTX from the vascular compartment to the tissues and results in non-linear pharmacokinetics and narrow distribution [80,85,87].

A phase 1 study (NCT03008512) of Genexol^®^-PM in patients with advanced malignancies [51] showed that the MTD was 390 mg/m^2^, higher than that of Abraxane^®^ (300 mg/m^2^) [53], with neuropathy, myalgia, and neutropenia as the main dose-limiting toxicities (DLTs) [51]. Furthermore, acute hypersensitivity reactions were not observed, despite no premedication taken [51]. The recommended dose (RD) for phase 2 studies was established as 300 mg/m^2^, higher than that of Taxol^®^ (175 mg/m^2^) in similar 3-week regimens [51]. At the phase 2 RD, Genexol^®^-PM was well tolerated and showed significant antitumor activity when administered as monotherapy for metastatic breast cancer [88], pancreatic cancer [89], and in combination with cisplatin for NSCLC [90], with an overall response rate (ORR) of 58.5%, 6.7%, and 37.7%, respectively. In the case of pancreatic cancer patients (NCT00111904), median overall survival (OS) and disease control rate (DCR) were improved when compared to gemcitabine monotherapy [89]. The most common AEs included neutropenia, fatigue, infection, dehydration, neuropathy, and abdominal pain [89]. A recent phase 1 study (NCT02739529) of weekly Genexol^®^-PM (100–120 mg/m^2^) combined with carboplatin (5–6 AUC) every 3 weeks for gynecologic cancer (adult solid tumor) showed an acceptable safety profile and an ORR of 72.2% [91]. 

Several phase 2 studies have demonstrated the efficacy and safety of Genexol^®^-PM in patients with advanced malignancies, including metastatic breast cancer [88], NSCLC [90], pancreatic cancer (NCT00111904) [89], and ovarian cancer (NCT01276548) [92]. Genexol^®^-PM (230 mg/m^2^) in combination with gemcitabine (1000 mg/m^2^) as first-line treatment for patients with advanced NSCLC (NCT01770795) demonstrated significant antitumor efficacy, with an ORR of 46.5% [93]. This combination regimen had lower rates of myelotoxicity and emetogenicity in comparison with the platinum-based doublet regimen of Genexol^®^-PM in NSCLC patients [90]. Weekly Genexol^®^-PM (100 mg/m^2^) combined with gemcitabine (1000 mg/m^2^) for treatment of unresectable or metastatic biliary tract cancer revealed an ORR of 25.6% and a DCR of 71.8%, without severe side effects [94]. Genexol^®^-PM (230 mg/m^2^) in combination with cisplatin (70 mg/m^2^) was highly effective and tolerable as first-line palliative chemotherapy of unresectable thymic epithelial tumors [95]. The ORR was 62.5% with rates of 70% for thymic carcinoma and 46% for advanced thymoma [95]. On the other hand, in locally advanced HNSCC, induction therapy with Genexol^®^-PM (230 mg/m^2^) and cisplatin (60 mg/m^2^) (NCT01689194) exhibited modest tumor response compared with the most effective regimen of DTX, cisplatin, and 5-fluorouracil, although with a more favorable toxicity profile and promising 3-year progression-free survival (PFS) (54.3%) and OS (71.3%) rates [96]. 

Genexol^®^-PM (240–300 mg/m^2^) as second-line chemotherapy in patients with advanced urothelial cancer after gemcitabine–cisplatin failure (NCT01426126) showed an ORR of 21% and a DCR of 65% [97]. These efficacy results were superior to second-line Taxol^®^ after prior platinum-containing regimens and compared favorably with those of Abraxane^®^ [97,98]. Moreover, Genexol^®^-PM monotherapy or in combination regimens allowed administration of higher PTX doses when compared with a conventional CrEL-based formulation (Genexol^®^), with lower incidence and severity of AEs, as observed in NSCLC [90,99], ovarian cancer [92,100], and urothelial cancer [97] patients. In a phase 3 study (NCT00876486) comparing the efficacy and safety of Genexol^®^-PM (260 mg/m^2^) with Genexol^®^ (175 mg/m^2^) for recurrent or metastatic HER2-negative breast cancer, Genexol^®^-PM demonstrated superior clinical efficacy and a manageable safety profile, with an ORR of 39.1% compared with 24.3% for the conventional PTX formulation [101]. A recently completed prospective cohort study (NCT05300828) to evaluate the safety profile of Genexol^®^-PM (280 mg/m^2^) plus carboplatin (AUC 5) as adjuvant therapy after cytoreductive surgery for newly diagnosed ovarian cancer patients showed that high-dose Genexol^®^-PM improved PFS compared to standard treatment (PTX 175 mg/m^2^ plus carboplatin AUC 5) and was as effective as addition of bevacizumab (15 mg/kg) to standard therapy, particularly in patients with stages III–IV high-grade serous carcinoma of the ovary who underwent optimal debulking surgery [102]. 

Genexol^®^-PM was the first polymeric micellar product in the market [83], being introduced in South Korea in 2007 as first-line therapy for breast cancer [92] and NSCLC (in combination with cisplatin) [90], and more recently approved as first line therapy for ovarian cancer [92] in combination with other chemotherapeutic agents [83]. Genexol^®^-PM is also sold in India, Vietnam, the Philippines, and Indonesia, being commercialized as Paxus™ in some Asian countries [83] while in the US and several countries of the European Union it is licensed under the name Cynviloq™ [83].

Preliminary pharmacokinetic data from the TRIBECA study (NCT02064829), a phase 3 clinical trial aiming at establishing bioequivalence between Cynviloq™ and Abraxane^®^, at 260 mg/m^2^, for patients with metastatic or locally recurrent breast cancer, supported potential for bioequivalence between the two formulations [75].

#### 4.1.2. Zisheng^®^

Zisheng^®^ (PM-Pac or PM-PTX) is another nanoparticle polymeric micellar PTX formulation for injection, also made from mPEG-*b*-PDDLA, independently developed by Shanghai Yizhong Pharmaceutical Co., Ltd. (Shanghai, China), and the first approved PTX-loaded PM in China [3]. Similarly to Genexol^®^-PM, Zisheng^®^ is CrEL-free and does not require premedication to prevent hypersensitivity reactions nor special non-PVC infusion systems or in-line filtration for administration, unlike a conventional CrEL-based formulation (Taxol^®^). The innovative PM-PTX dosage form showed improved efficacy and safety compared to a solvent-based (SB) PTX formulation (SB-PTX), both in vitro [103] and in vivo [103]. 

PM-PTX-induced apoptosis and cell viability inhibition in human NSCLC cell lines A549 and H226 in vitro was higher than that of SB-PTX [103]. In BALB/c nude mice, PM-PTX showed significantly enhanced tumor growth inhibition efficacy in the A549-derived xenograft tumor model when compared with SB-PTX at the same PTX dosage [103]. According to biodistribution studies, the ratios of PTX concentrations in major organ tissue to plasma concentrations were significantly higher in the PM-PTX group [55,103]. PM-PTX, with a size around 20 nm, passively targets tumor cells through the EPR effect and significantly reduces the retention time of PTX in the bloodstream, thus improving drug uptake and accumulation in tumor tissues [55,103]. Toxicity assessment and histopathological studies in healthy rats demonstrated that PM-PTX, at a 2–3-fold greater dosage than SB-PTX, significantly reduced the incidences of peripheral neuropathy, brain injury and liver damage, in terms of both short-term and long-term toxicity, but could induce potential male genital system toxicity (testicular and prostate atrophy) [103]. 

In a phase 1 study, patients with advanced solid malignancies (n = 18) received PM-PTX IV over 3 h, every 3 weeks, at escalating doses from 175 mg/m^2^ (level 1) to 435 mg/m^2^ (level 5), without acute hypersensitive reactions [55]. The ORR was 33.3% (including three patients with prior exposure to PTX chemotherapy), comparable with ORR for nab-PTX (Abraxane^®^) plus carboplatin (33%) [104] and for Genexol^®^-PM plus cisplatin (38%) [90] as first-line doublet regimens. All the patients treated with 435 mg/m^2^ PM-PTX developed DLT grade 4 neutropenia as well as one patient treated with a 300 mg/m^2^ dose (level 3). The incidence of neutropenia and peripheral sensory neuropathy became increasingly severe as the dose increased from 300 to 435 mg/m^2^. Thus, the MTD of PM-PTX was determined as 390 mg/m^2^ (level 4) while the recommended phase 2 dose was 300 mg/m^2^ [55], similarly to Genexol^®^-PM [51]. The formulation exhibited a linear pharmacokinetic profile, with the peak concentration and AUC values of PTX increasing with dosage [55], and a relatively longer half-life compared to Genexol^®^-PM [51] (Table 3).

A phase 3 study (NCT02667743) comparing the efficacy and safety between PM-PTX (230–300 mg/m^2^) plus cisplatin (70 mg/m^2^) and conventional SB-PTX (175 mg/m^2^) plus cisplatin (70 mg/m^2^) as first-line therapy for advanced NSCLC showed an improved ORR for the PM-PTX formulation (50% versus 26% for SB-PTX), observed in both squamous (59% versus 37%) and non-squamous (44% versus 19%) histological types, with significantly lower incidence of serious AEs [105]. Furthermore, the study provided clinical evidence that PM-PTX administration prolonged PFS and also OS with a favorable safety profile in NSCLC patients without pleural metastasis when compared with conventional SB formulation (SB-PTX) [106].

A recent retrospective study evaluating the efficacy and safety of PM-PTX (360 mg/m^2^) in combination with 5-fluorouracil (750 mg/m^2^) and leucovorin (200 mg/m^2^) as systemic chemotherapy for advanced gastric cancer demonstrated that the ORR was significantly higher than that of the conventional PTX (210 mg/m^2^) group (31% versus 10%), with lower incidence of anemia, leukopenia, liver dysfunction, nausea, vomiting, diarrhea, and allergy [107].

Several clinical trials of PM-PTX are currently ongoing or planned, either as monotherapy or in combination regimens for the treatment of solid tumors, including a phase 3 study (NCT06143553) evaluating PM-PTX for HER2-negative metastatic breast cancer in comparison with nab-PTX (Table 4). 

#### 4.1.3. Nanoxel^®^

Nanoxel^®^ is another CrEL-free PTX polymeric micellar formulation developed in India by Dabur Pharma, Ltd., Ghaziabad, Uttar Pradesh, India (later integrated into Fresenius Kabi Oncology Ltd., Himachal Pradesh, India), marketed in the country since 2006 [108] for metastatic breast cancer chemotherapy and later approved for the treatment of ovarian cancer, NSCLC, and AIDS-related Kaposi’s sarcoma [78]. The pH-sensitive PMs are composed of amphiphilic PVP-*b*-PNIPAM block copolymers [109]. The nanomicelles, with sizes in the range 80–100 nm, are stable at physiological pH (7.4) but in the acidic conditions of the TME, surface erosion of PNIPAM slowly releases PTX [109]. Due to their small sizes, the NPs passively target tumor cells by the EPR effect and selectively accumulate in the tumor, thus sparing normal tissue [110]. Contrary to Genexol^®^-PM and Abraxane^®^, which are lyophilized products that can be kept at room temperature, Nanoxel^®^ is a liquid formulation that requires storage at 2–8 °C [78]. 

Transmission electron microscopy (TEM) studies revealed that Nanoxel^®^ uptake in different human cancer cell lines, including NSCLC (A549), breast cancer (HBL-100), and ovarian cancer (PA-1) cells, is mediated by endocytosis, followed by intracellular drug release in the acidic endolysosomal compartment [110]. The intracellular drug uptake in Nanoxel^®^ was comparable to Abraxane^®^ and superior to a conventional CrEL-based PTX formulation (Intaxel^®^, Fresenius Kabi Oncology Ltd., Himachal Pradesh, India) in all the human cell lines tested, demonstrating the higher in vitro efficiency of the NP-based formulations for drug delivery to target cells [110]. 

A phase 1 dose escalation study evaluating a 3-weekly regimen of Nanoxel^®^ (135–375 mg/m^2^, administered as a 1 h infusion without premedication), for up to six cycles, in 23 patients with refractory or metastatic solid tumors showed that the formulation was well tolerated at a dose of 300 mg/m^2^, higher than that of conventional PTX, with no incidence of hypersensitivity reactions, febrile neutropenia, or neuropathies above grade 1 [56]. The MTD was determined at 375 mg/m^2^. Nanoxel^®^ showed a linear pharmacokinetic profile and promising antitumoral activity in advanced breast cancer, with 50% of cases of heavily pretreated breast cancer showing objective responses [56].

A subsequent phase 2, open-label study showed an ORR of 40% each in patients with advanced or metastatic breast cancer (after failure of anthracycline chemotherapy) that received Nanoxel^®^ (300 mg/m^2^ or 220 mg/m^2^) every 3 weeks for six cycles compared to 31% in patients treated with SB-PTX (Taxol^®^, 175 mg/m^2^) [111]. Neutropenia incidence was lowest in the Nanoxel^®^ 220 mg/m^2^ arm (39.4% versus 50% in the Taxol^®^ arm) but at the highest Naxoxel^®^ dosage (300 mg/m^2^), it was superior to Taxol^®^ (56.3%) [111]. Grade 3 sensory neuropathy occurred in 12.5% of patients receiving 300 mg/m^2^ Nanoxel^®^ compared with 1.5% and 6.3% of patients on Nanoxel^®^ 200 mg/m^2^ and Taxol^®^, respectively. Hypersensitivity reactions were not observed with Nanoxel^®^ despite the absence of premedication [111].

A retrospective study in a single hospital practice in India showed that in 84 cancer patients treated with Nanoxel^®^ there were no infusion reactions for a total of 596 infusions while other AEs, like hematological and gastrointestinal side effects, were similar to conventional PTX [108]. Neutropenia was more frequent in the Nanoxel^®^-treated group (22.68% versus 9.8%) while nausea (2.58% versus 9.8%) and vomiting (9.79% versus 21.57%) were more common in the conventional PTX group. The same study compared the OS between Nanoxel^®^ (n = 23) and conventional PTX (n = 28), in combination with a platinum agent, for the treatment of gastroesophageal cancer. The OS was 22 months for the Nanoxel^®^ group and 12 months for the conventional PTX group, but the difference was not statistically significant [108]. The 3-week cycle with Nanoxel^®^ was cost-effective when compared with conventional SB-PTX (at the same dosage and schedule regimen) regarding drug administration, necessity for premedication, and incidence and severity of AEs [108].

A more recent single center retrospective analysis suggested that a Nanoxel^®^–gemcitabine regimen was effective in advanced pancreatic cancer patients (n = 78) in routine clinical practice, with efficacy and toxicity similar to that of Abraxane^®^–gemcitabine at the same dosage (125 mg/m^2^ of Abraxane^®^ or Nanoxel^®^ followed by 1000 mg/m^2^ gemcitabine infusion on days 1, 8, and 15, every four weeks) [112]. Another longitudinal observational pharmacovigilance study conducted in a medical oncology ward in India over 18 months demonstrated that the adverse drug reaction profile of Nanoxel^®^ (n = 10) was statistically comparable to conventional PTX (n = 10) but suggested a better tolerability since a significantly higher dose (330 mg/m^2^ versus 260 mg/m^2^) was employed [113]. Common AEs included myalgia, nausea, anemia, paresthesia, alopecia, diarrhea, and vomiting while hypersensitivity reactions were not observed despite no premedication in the case of Nanoxel^®^ [113].

#### 4.1.4. Nanoxel^®^-M

Nanoxel^®^-M is a DTX polymeric micellar formulation developed by Samyang Biopharmaceutics Corporation (Seoul, South Korea) using the same amphiphilic diblock copolymer, mPEG-*b*-PDLLA, of their PTX micellar formulation, Genexol^®^-PM [81,83], similarly devoid of surfactant to prevent side effects commonly associated with Tween^®^ 80 used to solubilize DTX in the conventional formulation Taxotere^®^ that requires corticosteroid premedication.

In vitro cytotoxicity studies of Nanoxel^®^-PM in different human cancer cell lines, including H-460 (NSCLC), MCF-7 (breast cancer), and SKOV-3 (ovarian cancer), showed IC_50_ values comparable to the ones obtained for Taxotere^®^ (2.33 versus 4.66 ng/mL in H-460 cells, 1.73 versus 1.83 in MCF-7 cells, and 2.19 versus 3.25 ng/mL in SKOV-3 cells) [81]. In nude mice bearing human lung cancer (H-460) xenografts, IV administration of Nanoxel^®^-PM (13 mg/kg on days 0, 1, and 2) significantly delayed tumor growth and reduced tumor volume, showing comparable antitumor efficacy to Taxotere^®^ [81]. The dose of Nanoxel^®^-PM chosen, 13 mg/kg, corresponded to 117 mg/m^2^/cycle, close to the highest recommended single-agent dose of Taxotere^®^ (100 mg/m^2^) in humans [81].

Pharmacokinetic studies in mice, rats, and beagle dogs revealed similar pharmacokinetic profiles between Nanoxel^®^-PM and Taxotere^®^. Moreover, the relative magnitudes of AUC_0→∞_ and *C*_max_ of Nanoxel^®^-PM compared to those of Taxotere^®^ were within 100% ± 20%, demonstrating bioequivalence [81]. The similarity in the pharmacokinetic profile of both formulations was attributed to DTX release from the micelles to bind albumin plasma protein after IV administration [81]. 

However, Nanoxel^®^-M enhanced collagen-induced (but not thrombin-induced) rat platelet aggregation in vitro while Taxotere^®^ inhibited it, suggesting that the micellar formulation altered the toxicological profile of DTX [114]. Toxicity studies showed that a single IV infusion (30 min) of Nanoxel^®^-PM or Taxotere^®^ to rats or three daily IV (bolus) administrations to mice at low (10 mg/kg), intermediate (13 mg/kg), and high (15 mg/kg) doses produced no significant differences in body weight changes or white blood cell (WBC) counts between the two treated groups at all tested doses [81]. A dose-escalating single IV toxicity study of Nanoxel^®^-PM in beagle dogs with three ascending doses (0.25, 0.50, and 0.75 mg/kg) did not detect any hypersensitivity reactions or fluid retention, contrary to Taxotere^®^ administration, which was accompanied by severe anaphylactic-type reactions (e.g., erythema, facial swelling, and dyspnea) at all dose levels despite premedication with dexamethasone [81]. Furthermore, plasma histamine levels were related to the onset and duration of hypersensitivity reactions in the Taxotere^®^ group, which showed a much higher peak level compared with Nanoxel^®^-PM (75–132 ng/mL versus 0.4–2.2 ng/mL). The infusion reactions to Taxotere^®^ were attributed to the presence of Tween^®^ 80 and suggest an improved safety profile for the surfactant-free Nanoxel^®^-PM formulation.

A multicenter trial to evaluate the safety and toxicity of Nanoxel^®^-M as adjuvant therapy, alone or in combination with cyclophosphamide, after surgery for early breast cancer in Korean patients (n = 55) showed that the micellar formulation reduced the incidence of taxane-induced peripheral neuropathy and thrombocytopenia compared with Taxotere^®^ without vehicle-associated hypersensitivity reactions [115]. The most common side effects were grade 3/4 neutropenia (61.8%) followed by febrile neutropenia (4.5%) and mucositis (1.4%) [115]. 

A phase 2 multicenter study of Nanoxel^®^-PM and trastuzumab-pkrb (biosimilar to trastuzumab, a mAb against HER2) combination therapy in HER2-positive advanced salivary duct carcinoma demonstrated promising antitumor activity with a manageable toxicity profile [116]. Patients (n = 43) treated with Nanoxel^®^-PM (75 mg/m^2^) and trastuzumab-pkrb (8 mg/kg in the first cycle and 6 mg/kg in subsequent cycles) every 3 weeks showed an ORR of 69.8%, DCR of 93.0%, median PFS of 7.9 months, and median OS of 23.3 months [116]. The most common treatment-related AEs were peripheral edema, myalgia, stomatitis, and alopecia while grade 3/4 AEs included neutropenia, febrile neutropenia, anemia, and decreased left ventricular ejection fraction, the latter related to trastuzumab-pkrb [116].

A multicenter, prospective observational study (NCT04066335) is currently ongoing to evaluate the safety of Nanoxel^®^-M injection in patients with breast cancer, NSCLC, and prostate, ovarian, head and neck, gastric, or esophageal cancers. 

#### 4.1.5. NK105

NK105 is another freeze-dried PTX polymeric micellar formulation developed by NanoCarrier Co., Ltd. (Chiba, Japan) and licensed to Nippon Kayaku Co., Ltd. (Tokyo, Japan), composed of the amphiphilic block copolymers PEG and poly(aspartate), the latter modified by esterification with 4-phenyl-1-butanol to increase hydrophobicity of the core [117]. The PMs, with a size around 85 nm, allowed high drug loading (23% *w*/*w* PTX) through passive entrapment of the drug in the micellar core via hydrophobic interactions and provided effective drug retention following IV administration [117]. 

A preclinical pharmacokinetic study on colon 26 tumor-bearing CDF1 mice found that the plasma and tumor AUC values were around 90- and 25-fold higher, respectively, for NK105 than for free PTX after a single IV injection (100 mg/kg) of the drugs, due to prolonged circulation in the bloodstream and the EPR effect associated with the micellar formulation [117]. Moreover, at 72 h after the IV injection, the tumor PTX concentration was above 10 μg/g in the NK105 group but less than 0.1 μg/g in the free PTX group. 

Although NK105 and conventional PTX formulation showed equivalent cytotoxic activity in vitro, exhibiting similar dose–response curves and IC_50_ values on several human cancer cell lines derived from lung, gastric, esophagus, colon, breast, and ovarian tumors, NK105 showed improved in vivo antitumor efficacy in nude mice bearing human colorectal cancer (CRC) HT-29 xenografts due to enhanced tumor exposure via the EPR effect and sustained release from the micellar NPs [117]. Furthermore, repeated administration of NK105 to rats at 7-day intervals showed attenuated peripheral neurotoxicity when compared with free PTX [117], which was attributed to NK105 exclusion from the rat dorsal root ganglion (DRG) due to particle size (around 85 nm), while albumin-bound PTX particles of around 8 nm formed after injection of SB-PTX formulation can extravasate into DRG parenchyma, consistent with subsequent pharmacokinetic and histopathological studies [118].

NK105 (45 mg/kg single IV injection) was also a more potent radiosensitizing agent compared to free PTX at the same dosage in Lewis-lung-carcinoma-bearing mice due to more severe cell cycle arrest at the G_2_/M phase induced by NK105 [119]. Histopathological examination of the mice lung sections revealed inflammatory cell infiltration, the presence of intra-alveolar macrophages, and destruction of the alveolar architecture, which were due to thoracic radiation and not to NK105 accumulation in the lung [119].

A phase 1 and pharmacokinetic study of NK105 (10–180 mg/m^2^), administered to cancer patients (n = 19) as a 1 h IV infusion, every 3 weeks, without antiallergic premedication showed that NK105 was well tolerated, and the RD for the phase 2 study was established as 150 mg/m^2^ every 3 weeks [57]. The DLTs included grade 4 neutropenia and grade 3 fever at the 180 mg/m^2^ dose, which was designated as the MTD. NK105 exhibited a linear pharmacokinetic profile and its plasma AUC at 150 mg/m^2^ was nearly 15-fold higher compared with that of the conventional PTX formulation at 210 mg/m^2^ (conventional dose for a 3-week regimen in Japanese patients), consistent with the stability of the micelle formulation in plasma [57]. 

A phase 2 study to evaluate the efficacy and safety of NK105 (150 mg/m^2^ IV infusion for 30 min every 3 weeks) in patients with advanced or recurrent gastric cancer (n = 57) after the failure of first-line chemotherapy showed modest antitumor activity, with an ORR of 25% and median PFS and OS of 3.0 and 14.4 months, respectively [120]. The most common AEs were alopecia, peripheral neuropathy, fatigue, myalgia, anorexia, rash, arthralgia, stomatitis, diarrhea, and nausea. Grade 4 toxicities included neutropenia (64.9%), leukocytopenia (17.5%), anemia (12.3%), lymphopenia (8.8%), and peripheral neuropathy (1.8%) but no grade 3/4 hypersensitive reactions were observed [120]. 

However, a phase 3 clinical trial (NCT01644890) comparing NK105 (65 mg/m^2^ on days 1, 8, and 15 of a 28-day cycle) and PTX (80 mg/m^2^, same schedule) in metastatic or recurrent breast cancer (n = 436) missed its primary endpoint (PFS with a non-inferiority margin of 1.215) [121]. The micellar formulation provided an ORR of 31.6% and median OS of 31.2 months compared with 39.0% and 36.3 months for PTX, respectively, although NK105 exhibited a more favorable toxicity profile, with lower incidence of grade 3/4 peripheral sensory neuropathy (1.4% versus 7.5% for PTX) [121]. 

Another phase 1 study to determine the RD of weekly administered NK105 (50–100 mg/m^2^ IV infusion over 30 min) for 3 consecutive weeks in each 4-week cycle in patients with solid tumors (n = 16) found DLTs at 100 mg/m^2^ due to neutropenia and the RD was established as 80 mg/m^2^ [58]. In the subsequent exploratory dose-expansion phase, six out of ten patients treated with weekly NK105 at the RD achieved partial response and four reached stable disease status [58]. Neutropenia of grade ≥ 3 occurred in eight patients, requiring dose reduction or dose delay. On the other hand, non-hematological events, namely peripheral sensory neuropathy, were mostly grade 1, and no hypersensitivity reactions were observed. Based on these results, an initial NK105 dose of 65 mg/m^2^, lower than the RD (80 mg/m^2^) determined in the dose-escalation phase, was selected for the ensuing phase 3 study. 

A phase 2 study comparing NK105 and PTX in advanced or recurrent breast cancer (n = 123), with both drugs being intravenously administered at 80 mg/m^2^ on days 1, 8, and 15 of a 28-day cycle, revealed no significant difference in ORR, median PFS, and OS between the two groups [122]. The incidence of hematologic AEs was higher in the NK105 group, namely neutropenia (79.0% versus 55.7%), with several patients requiring treatment with granulocyte-colony-stimulating factor (G-CSF). However, the incidence of peripheral sensory neuropathy was lower in the NK105 group (64.5% versus 82.0%) with no grade 3/4 non-hematologic events [122]. 

A recent systematic review and meta-analysis comparing the efficacy and peripheral neuropathy of SB-PTX with NK105 monochemotherapy revealed no significant differences between the incidence of all-grade peripheral neuropathy among both groups [123]. However, the incidence of high-grade peripheral neuropathy was lower in the NK105 group, which also showed longer OS in cancer patients [123]. 

#### 4.1.6. CriPec^®^ Docetaxel

CriPec^®^ Docetaxel (CPC634), developed by Cristal Therapeutics (Maastricht, Netherlands), is a DTX-incorporating core-crosslinked PM with 65 nm size composed of mPEG_5k_ and thermosensitive *N*-(2-hydroxypropyl)methacrylamide-oligolactate block copolymers (mPEG_5k_-*b*-pHPMA-Lac_n_) based on CriPec^®^ technology, with DTX covalently bound to the crosslinked core through a hydrolyzable ester linker [124].

The CriPec^®^ platform (Cristal Therapeutics, Netherlands) is based on amphiphilic block copolymers made of hydrophilic mPEG and thermosensitive *N*-(2-hydroxypropyl)methacrylamide (HPMA) derivatized with lactate side chains, designed to covalently entrap active pharmaceutical ingredients in CCPMs upon self-assembly [124]. Partial esterification of the lactate side chains of the hydrophobic segment with methacrylic acid allows covalent crosslinking of the hydrophobic blocks forming the micellar core by free radical polymerization, resulting in improved micelle stability and avoiding premature drug leakage. Furthermore, covalent attachment of the drug to the micellar core by free radical polymerization upon drug functionalization by covalent conjugation with a biodegradable linker containing a polymerizable moiety allows control of the site and rate of drug release by appropriate choice of the linker and enables higher encapsulation efficiency compared with physical loading [124]. The reactive block copolymers and the drug-linkers self-assemble into a micellar structure with the drug physically encapsulated in the micellar core, and the hydrophobic core-forming block copolymers and drug-linker are cocrosslinked by free radical polymerization, forming a 3D network [124]. Tunable size, within the range 35–100 nm, is dependent on the MW of the block copolymers [124]. The stealth effect of the hydrophilic dense PEG shell provides prolonged circulation while core-crosslinking, by preventing the reorganization of micelles, further contributes to reducing the interaction with plasma proteins. Neglectable protein corona was observed for CPC634 when incubated in human blood plasma [125].

CPC634 is manufactured as an aqueous dispersion stable for at least 5 years when stored at −80 °C to prevent premature drug release and hydrolysis of the core-crosslinks [124]. To overcome cold chain supply problems, a lyophilization methodology using trehalose as a cryoprotectant yielded a stable CPC634 freeze-dried cake with a moisture content lower than 0.1 wt% [126]. The trehalose-cryoprotected CPC634 could be reconstituted in less than 5 min at room temperature, with size, morphology, drug retention, and release kinetics identical to those of the non-freeze-dried formulation, and the methodology is readily translatable to large-scale manufacturing [126].

The covalent conjugation of DTX to the crosslinked core of CPC634 micelles allowed for in vitro sustained drug release under physiological conditions (PBS pH 7.4, 37 °C) upon hydrolysis of the ester linker and followed first-order kinetics [127]. A similar in vitro drug release profile was also observed in whole human blood at 37 °C, corroborating that DTX release from CPC634 is driven by chemical hydrolysis, since the crosslinked micellar core prevents enzyme access [127]. 

In mice bearing human breast (MDA-MB-231) tumor xenografts, administration of CPC634 (30 or 60 mg/kg single injection in tail vein) showed superior therapeutic efficacy compared to the marketed DTX formulation (Taxotere^®^) at the same dose [127]. Furthermore, a single IV injection of CPC634 at 125 mg/kg was enough to achieve complete regression of both small (150 mm^3^) and established (550 mm^3^) tumors, resulting in 100% survival of the animals. The potent antitumor effects of the nanoformulation were attributed to enhanced tumor accumulation and antistromal activity [127]. CPC634 also displayed better tolerability in healthy rats compared to Taxotere^®^ [127].

A study of empty CPC634 CCPMs decorated with the cyclic RGD (cRGD) peptide targeting α_v_β_3_ integrins has also been performed, showing higher in vitro uptake in cell lines expressing high levels of α_v_β_3_ (e.g., A431 epidermoid carcinoma cells) [128]. In these cells, the cRGD-CPPMs were more efficiently internalized than the non-functionalized CPPMs (control), being found in the perinuclear region while peptide-free CCPMs colocalized with endosomes/lysosomes [128]. The uptake of cRGD-CCPM was not proportional to the increase in cRGD decoration, suggesting that relatively low decoration densities (1 mol% cRGD) may be enough for CCPM targeting and uptake in vivo without affecting their pharmacokinetic and biodistribution profiles [128].

A first-in-human phase 1, dose-escalation, and pharmacokinetic study (NCT02442531, NAPOLY trial) of CPC634 in patients with advanced solid tumors (n = 33) receiving CPC634 intravenously every 3 weeks (15–100 mg/m^2^), every 2 weeks (45 mg/m^2^) or every 3 weeks (60 mg/m^2^) with dexamethasone premedication showed that cumulative skin toxicity at doses ≥ 60 mg/m^2^ was the main DLT, which was absent in the corticosteroid-pretreated group [59]. Thus, the recommended phase 2 dose was determined at 60 mg/m^2^ every 3 weeks with dexamethasone premedication. The formulation exhibited a dose-proportional pharmacokinetic profile with prolonged systemic exposure to DTX, in accordance with preclinical studies [59]. The development and validation of a bioanalytical method for the determination of both total and released DTX from CPC634 in human plasma and tumor tissue using sensitive and selective liquid chromatography–tandem mass spectroscopy (LC-MS/MS) were successfully applied in the pharmacokinetic analysis of serum and tissue samples from cancer patients treated with CPC634 [129]. 

A two-arm pharmacokinetic study (CriTax study) in patients with solid tumors (n = 24) randomized to receive CPC634 (75 mg/m^2^, 1 h IV infusion) in cycle 1 and conventional DTX (75 mg/m^2^, 1-h IV infusion) in cycle 2 (arm A) or vice versa (arm B) revealed that the plasma AUC was 27% higher for CPC634-released DTX while *C*_max_ was 91% lower compared with conventional DTX, which contributed to a lower incidence of neutropenia during CPC634 treatment [60]. Tumor biopsies showed that CPC634 administration enhanced the intratumoral DTX exposure, resulting in 4.6-fold higher total DTX concentration in the metastatic lesions compared with conventional DTX but comparable released DTX concentration [60]. 

Additionally, CPC634 administration resulted in a 3.7-fold higher total skin DTX concentration compared with conventional DTX while the released DTX concentrations were not statistically different [130]. Histopathological examination of skin biopsies taken at baseline and at day 8 of both cycles revealed increased apoptosis and micronucleation after treatment with either CPC634 or conventional DTX, which could induce inflammatory reactions leading to skin toxicity, often associated with DTX treatment [130].

A phase 2 study (NCT03742713, CINOVA trial) of CPC634 in 24 patients with platinum-resistant recurrent ovarian cancer showed disappointing clinical activity of the formulation [131]. None of the patients had an objective response, and the trial was prematurely stopped due to futility [131]. The most common AEs were mainly gastrointestinal (96%) but also fatigue (44%), dyspnea (40%), and infections (40%) [131].

A first-in-human imaging study (NCT03712423, PICCOLO trial) with zirconium-89-radiolabeled CPC634 was performed to enable visualization and quantification of NP accumulation in human solid tumors [132]. In seven patients with solid tumors administered ^89^Zr-CPC634 at a high therapeutic dose (60 mg/m^2^ DTX) or a low diagnostic dose (1–2 mg DTX), positron emission tomography–computed tomography (PET/CT) imaging showed accumulation in 46% and 41% of tumor lesions, respectively, and pharmacokinetic mean half-life of 97.0 ± 14.4 h for the therapeutic dose and 62.4 ± 12.9 h for the diagnostic dose [132]. Thus, PET/CT imaging with a diagnostic dose of ^89^Zr-CPC634 accurately reflects tumor accumulation of the therapeutic dose without causing any AEs, showing potential for patient stratification in clinical practice [132].

### 4.2. Irinotecan-Based Micelle Formulations

Irinotecan (CPT-11) is a water-soluble semi-synthetic derivative of the natural alkaloid camptothecin isolated from the bark and stem of the Chinese tree *Camptotheca acuminata*, which is frequently used in the chemotherapy of advanced or metastatic CRC [133]. In vivo, the CPT-11 prodrug is hydrolyzed by carboxylesterases into the pharmacologically active metabolite 7-ethyl-10-hydroxycamptothecin (SN-38), which is metabolized in the liver into the inactive glucuronide by hepatic uridine 5’-diphosphoglucuronosyl transferase 1A1 (UGT1A1) and 1A7 isomorphs (UGT1A7) and mainly excreted in the bile [133]. In the intestine, SN-38G suffers deglucuronidation by bacterial β-glucuronidases, regenerating SN-38, which can be reabsorbed, resulting in the enterohepatic recirculation of SN-38. Late-onset diarrhea experienced by a vast majority of cancer patients on irinotecan-based chemotherapy has been attributed to intestinal overexposure to SN-38, the major active metabolite of irinotecan [133]. Additionally, polymorphisms of the UGT1A1 gene (e.g., UGT1A1*6 and UGT1A1*28), have been associated with a higher risk of severe neutropenia and irinotecan-induced delayed diarrhea [134]. 

SN-38, like camptothecin and its derivatives, is an inhibitor of DNA topoisomerase I (Top1), a nuclear enzyme involved in DNA replication and transcription highly expressed in cancer tissues, forming a stable drug–enzyme–DNA ternary complex that hinders DNA replication, ultimately resulting in apoptosis and cell death [133]. Contrary to CPT-11, SN-38 is poorly water-soluble (<5 μg/mL) and unstable at pH > 6 due to spontaneous and reversible hydrolysis of its lactone ring to the inactive carboxylate open-ring form [133].

### NK012

NK012, developed by NanoCarrier Co., Ltd. (Chiba, Japan) and licensed to Nippon Kayaku, Co., Ltd. (Tokyo, Japan), is a freeze-dried polymeric micellar formulation of SN-38, the pharmacologically active metabolite of CPT-11. NK012 micelles, with a 20 nm diameter, are prepared by self-assembly of amphiphilic diblock copolymers made of PEG_12k_ and p(Glu)_7k_ bearing 20% (*w*/*w*) SN-38 covalently attached to the carboxylate groups of the hydrophobic amino acid segment by ester bonds [133,135]. Unlike CPT-11, NK012 can release SN-38 through chemical hydrolysis of the phenyl ester bond at physiological pH (pH 7.4), thus its therapeutic effect is independent of carboxylesterase enzymatic activity, which varies among the population [133,135]. In vitro release studies showed that the amount of SN-38 released from NK012 in PBS (pH 7.3) at 37 °C achieved 57% at 24 h and 74% at 48 h and that these values decreased to 1% and 3%, respectively, in 5% glucose solution at pH 4.6, demonstrating the stability of the formulation in weak acidic media [135]. 

Extensive preclinical studies demonstrated the potent antitumoral activity of NK012 in vivo, particularly against solid tumors, being more effective than the SN-38 prodrug CPT-11 by selectively accumulating in the tumor tissue via the EPR effect and exhibiting a safer intestinal toxicity profile in several human tumor xenografts [133,135]. Moreover, the small size (20 nm) of NK012 also allowed effective penetration and distribution within hypovascular and stroma-rich tumors, like pancreatic cancer [136,137] and scirrhous stomach cancer [138], often intractable due to inefficient penetration of anticancer agents. In this regard, orthotopic tumor xenografts provide a better model compared with subcutaneous xenografts in terms of tumor vascularity and intersticium [137].

NK012 (30 mg/kg/day) efficacy in mice bearing orthotopic human pancreatic cancer cell (SUIT-2) xenografts was superior to that of CPT-11 (66.7 mg/kg/day) and gemcitabine (16.5 mg/kg/day), reducing the number of metastatic nodules in the peritoneal cavity, due to enhanced accumulation within the tumor tissue and sustained release of SN-38 from NK012 [137]. In mice orthotopically transplanted with scirrhous gastric cancer cells, NK012 showed enhanced distribution with prolonged SN-38 release when compared with CPT-11 and was effective against peritoneal nodules [138]. 

In mice bearing metastasis to the liver, colonized 7 days after portal vein administration of human colon cancer HT-29 cells, NK012 administration (30 mg/kg) eradicated the liver metastasis and improved survival rate compared with CPT-11 (66.7 mg/kg) [139]. Prolonged accumulation of NK012 and free SN-38 released from the PMs was observed in the tumors, liver, and spleen, lasting for 6 weeks after NK012 administration, while accumulation of free SN-38 converted from the CPT-11 prodrug rapidly decreased within 24 h [139]. Similarly, NK012 displayed a stronger antitumor effect compared with CPT-11 against liver metastasis produced by injecting human gastric cancer HSC-57 cells into the portal vein of mice, with a survival rate of 100% on day 131 versus 0% in the CPT-11-treated group [140]. 

NK012 showed significantly higher antitumor activity in nude mice with human CRC HT-29 cell xenografts subcutaneously implanted when compared with CPT-11 [141]. Pharmacokinetic analysis revealed that the plasma AUC of NK012 (30 mg/kg) was nearly 200-fold higher than that of CPT-11 (66.7 mg/kg) while the AUC of free SN-38 released from NK012 was 14-fold higher than that obtained from CPT-11 [141]. Moreover, the tumor concentration of free SN-38 reached 90.4 ng/g and 4.5 ng/g at 24 h after administration of NK012 or CPT-11, respectively, suggesting that prolonged circulation of NK012 in the bloodstream enhances tumor distribution due to the EPR effect, resulting in potent antitumor activity of NK012 in vivo [141]. Similarly, in combination therapy regimens with 5-fluorouracil, the replacement of CPT-11 by NK012 resulted in a higher synergistic antitumor effect in this experimental model of human CRC [142]. 

In the subcutaneous murine syngeneic renal adenocarcinoma (Renca) model, used as a hypervascular tumor model mimicking human renal cell carcinoma, NK012 (20 mg/kg/day on days 0, 4, and 8) was able to eradicate fast-growing Renca tumors in 60% of mice [143]. Injection of Renca cells into the tail vein of mice resulted in lung metastases, but treatment with NK012 significantly reduced the number of metastatic nodules and improved survival [143]. Biodistribution studies revealed an enhanced and prolonged distribution of free SN-38 in metastatic lung tissues, but not in healthy (non-metastatic) lung tissue, after NK012 administration [143].

In mice subcutaneously injected with VEGF-secreting human small-cell lung cancer cells (SBC-3/VEGF), NK012 markedly enhanced SN-38 distribution and accumulation in tumors due to the EPR effect, promoted by the hypervascularity and hyperpermeability induced by VEGF, resulting in remarkably higher antitumor activity when compared with CPT-11 [141]. Similarly, combination therapy with NK012 and cisplatin provided superior efficacy in relative tumor volume reduction compared with CPT-11/cisplatin and was not associated with severe diarrhea [144]. The higher concentration of CPT-11 found in the small intestine epithelium, which can be reabsorbed and converted to SN-38 that damages the intestinal mucosa and provokes diarrhea, was responsible for intestinal toxicity in the CPT-11/cisplatin-treated group [144]. NK012 in combination with S-1, an oral dihydropyrimidine dehydrogenase inhibitory fluoropyrimidine, also displayed a synergistic efficacy superior to that of CPT-11/S-1 and reduced intestinal toxicity, including lower incidence of diarrhea, in mice subcutaneously implanted with NSCLC (PC-14 or EBC-1 cell) xenografts [145].

Therapeutic combination of NK012 (5 or 30 mg/kg) with bevacizumab (5 mg/kg), an anti-VEGF humanized mAb, was more efficient than NK012 (5 or 30 mg/kg) at inhibiting tumor growth in nude mice subcutaneously implanted with human lung cancer (PC-14 or A549) xenografts [146]. Pharmacokinetic data revealed that the concentrations of NK012 and free SN-38 after administration of NK012 alone were not significantly different from those obtained for the combination of NK012 plus bevacizumab, suggesting that VEGF-induced angiogenesis inhibition by bevacizumab does not disturb NK012 tumor accumulation and produces an additional antitumor effect by reducing the area of proliferating vascular endothelial cells in the tumors [146].

Glioma is another type of hypervascular tumor with irregular vascular architecture and high expression levels of VEGF. NK012 was more effective than CPT-11 at reducing tumor volume and increasing survival rate in mice bearing glioblastoma (U87MG) orthotopic xenografts, which was attributed to enhanced intratumoral accumulation of NK012 with prolonged and sustained release of SN-38, since SN-38 antitumor activity is time-dependent [147]. However, free SN-38 was not detected in normal brain tissues after IV injection of either NK012 or CPT-11, suggesting that both NK012 and CPT-11 are unable to cross the BBB in the normal brain but extravasate from the brain tumor blood vessels, with the NPs preferentially accumulating in the tumor tissue [147]. Further studies showed that NK012 monotherapy was even more effective against orthotopic tumors than CPT-11 combined with bevacizumab [148]. Moreover, convection-enhanced delivery (CED) of NK012 enabled consistent distribution of SN-38 with minimum brain tissue damage in healthy rat brains after delivery of 40 μg NK012 while severe damage was observed with SN-38 at the same dose [149]. CED circumvents the BBB by delivering agents directly into the tumor or surrounding brain parenchyma based on continuous positive-pressure infusion, resulting in large volumes of distribution and high local drug concentrations with reduced potential systemic toxicity [149]. Local delivery of NK012 via CED significantly prolonged survival in rats with human U87MG brain tumor orthotopic xenografts [149].

In a mouse model of orthotopic multiple myeloma created using CD138-positive U2661B1 cells, which produce human IgE lambda light chain (monoclonal protein), IV administration of NK012 was able to suppress plasma elevation of human monoclonal protein levels and proliferation of CD138-positive myeloma cells in mouse bone marrow in a dose-dependent manner [150]. NK012 monotherapy and in combination with the proteasome inhibitor bortezomib prolonged the median survival time compared with the control (untreated) group and bortezomib alone, respectively [150]. 

A first-in-human phase 1, dose-escalating study in Japan, enrolling patients with solid tumors refractory to standard therapy (n = 24), showed that administration of NK012 (2–28 mg/m^2^ as a 30 min IV infusion every 3 weeks) was well tolerated and objective responses were observed in patients with refractory esophageal cancer and lung carcinoma [62]. The most common DLT was neutropenia, observed in two out of nine patients at the 28 mg/m^2^ dose level during cycle 1. Non-hematologic toxicity, namely diarrhea, was mostly grade 1/2. A subsequent phase 2 study evaluating the efficacy and safety of NK012 (28 mg/m^2^ IV infusion over 30 min, every 3 weeks) in Japanese patients with unresectable metastatic CRC (n = 58) previously treated with an oxaliplatin-based chemotherapy regimen found similar ORRs between NK012 monotherapy and irinotecan (CPT-11) monotherapy (3.8% versus 4.2%, respectively) reported in the phase 3 EPIC trial but with low incidence of grade ≥ 3 diarrhea [151]. The study included cancer patients homozygous or heterozygous for UGT1A1*28 or UGT1A1*6 originally excluded in the phase 1 trial. Based on the incidence and severity of grade ≥ 3 neutropenia and febrile neutropenia, the initial dose of 28 mg/m^2^ NK012 was considered too high for these patients. 

Another phase 1 dose escalation study was independently conducted in the USA [63]. Administration of NK012 (9–37 mg/m^2^ as a 30 min infusion, every 21 or 28 days, without premedication) to patients with previously treated advanced solid tumors (n = 38) showed promising antitumor activity, with partial responses in triple-negative breast cancer (n = 3), SCLC (n = 1), endometrial cancer (n = 1), and pancreatic neuroendocrine tumor (n = 1). The recommended phase 2 dose was set as 20 mg/m^2^ every 28 days, which was also identified as the MTD in the 21-day schedule, with myelosuppression as the main DLT [63]. Gastrointestinal toxicity was mild, including grade < 3 diarrhea. Pharmacokinetic analysis showed that NK012 (28 mg/m^2^) had a higher plasma AUC compared to that of CPT-11 (250 mg/m^2^) and that the half-life of SN-38 was significantly prolonged in NK012 when compared to CPT-11, demonstrating a sustained high systemic concentration of SN-38 in the micellar formulation [63]. 

A phase 1/2 study of NK012 (12–24 mg/m^2^) in patients with relapsed or refractory multiple myeloma (n = 16) established 20 mg/m^2^ as the RD of NK012, with grade 4 neutropenia being responsible for the majority of DLTs at a dose of 24 mg/m^2^ [152]. However, the study was terminated at the end of the phase 1 stage since all patients failed to achieve an objective response [152].

### 4.3. Anthracycline Micellar Formulations

Anthracyclines are antibiotics produced by *Streptomyces* spp. with a broad spectrum of antitumoral activity. Chemically, anthracyclines are glycoside drugs consisting of an anthraquinone aglycone coupled with an amino sugar (daunosamine), and the intercalation of the planar aromatic anthraquinone moiety between adjacent DNA base pairs contributes to their cytotoxic effects. Although anthracyclines are DNA-intercalating agents, inhibition of eukaryotic topoisomerase II (Top2) in proliferating cancer cells is considered the primary mode of action responsible for the potent cytotoxic activity of the drugs [153]. Anthracycline antibiotics are Top2 poisons that trap the enzyme–DNA cleavage complexes by stacking between DNA base pairs at the DNA/protein interface, interacting with both nucleotides as well as amino acid residues to form stable ternary complexes, which inhibits DNA re-ligation, generating DNA double-strand breaks and triggering apoptotic cell death [153]. 

Doxorubicin (DOX) is a cytotoxic anthracycline antibiotic produced by *Streptomyces peucetius* subsp. *caesius* (ATCC 27952) with a characteristic red color and natural fluorescence due to the anthraquinone chromophore. DOX is used alone or in combination with other chemotherapeutic agents as first-line therapy for several types of cancer, including breast, ovarian, thyroid, bladder, SCLC, bone sarcomas, neuroblastoma, acute lymphoblastic/myeloblastic leukemia, and Hodgkin lymphoma [153,154]. However, the therapeutic effect of DOX is limited by severe AEs, namely myelosuppression, nephrotoxicity, and dose-dependent acute and chronic cardiotoxicity. Among those, the most deleterious side effect is cardiomyopathy, potentially leading to congestive heart failure [153,154]. 

Besides being a Top2 poison and a DNA-intercalating agent, DOX-induced intracellular ROS generation also contributes to the drug cytotoxicity. At physiological pH, the amine sugar moiety of DOX is positively charged and binds with high affinity to negatively charged cardiolipin present in the inner mitochondrial membrane of metabolically active cells (e.g., cardiomyocytes and hepatocytes), promoting DOX accumulation in these organelles. DOX is reduced by microsomal (NADPH-cytochrome P450) and mitochondrial (Complex I) oxidoreductases to a semiquinone radical species, which can complex with Fe^2+^. The free radical complex can spontaneously reduce oxygen to superoxide anion radical, regenerating DOX in the process and reinitiating the cycle [153]. Increased levels of superoxide and other ROS and RNS generated in the process, including hydroxyl radicals and peroxynitrite, contribute to oxidative and nitrosative stress, mitochondrial dysfunction, DNA damage, and lipid peroxidation-dependent ferroptosis [153]. Although ROS detoxification can be achieved by endogenous antioxidative enzymes, such as superoxide dismutase (SOD), catalase, and glutathione peroxidase, the lower levels of these free-radical-scavenging systems expressed in cancer and myocardial cells contribute to DOX antitumor activity and associated cardiotoxicity [153]. Doxorubicinol (DOXol), a cytotoxic metabolite formed by the reduction of DOX catalyzed by cytosolic NADPH-dependent carbonyl and aldo-keto reductases located in erythrocytes, heart, liver, and kidney cells, disrupts calcium homeostasis by interfering with the sarcoendoplasmic reticulum calcium ATPase (SERCA) and the cardiac ryanodine receptor (RyR2) and is a relevant contributor to DOX-induced cardiomyopathy [153].

DOX is available in the form of a water-soluble salt, DOX hydrochloride (Adriamycin^®^, Farmitalia-Carlo Erba, Milan, Italy), for IV administration, usually with a cardioprotective agent, such as dexrazoxane, an iron chelator [154]. Liposomal formulations for injection (e.g., Doxil^®^/Caelyx^®^, Myocet^®^) are also available with lower incidence of cardiotoxicity but more expensive [154]. Furthermore, the pegylated liposomal formulation of DOX (Doxil^®^/Caelyx^®^) is associated with dose-limiting palmar plantar erythrodysthesia [154]. 

Several anthracycline analogs have been synthesized in attempts to improve therapeutic efficacy and reduce off-target toxicity. Among the few that reached the market was epirubicin (4’-epidoxorubicin), the 4’-epimer of DOX. Epirubicin (EPI) and DOX have similar potency, but EPR exhibits reduced cardiotoxicity, which is reflected in their maximum recommended cumulative doses, 1000 mg/m^2^ for EPR and 550 mg/m^2^ for DOX [83]. 

#### 4.3.1. NK911

NK911, developed by NanoCarrier Co., Ltd. (Chiba, Japan) and licensed to Nippon Kayaku (Tokyo, Japan), is a polymeric micellar formulation of DOX with a 40 nm size composed of amphiphilic diblock copolymers made of PEG_5k_ and p(Asp)_4k_, made more hydrophobic by partial conjugation (near 45%) of the drug to the carboxylic groups of the amino acid side chains through amide bonds, and containing physically entrapped DOX stabilized via π–π-stacking interactions with the conjugated drug [155]. Contrary to the ester bonds in NK012, the amide bond is hydrolytically stable, and it is the physically encapsulated free drug rather than conjugated DOX that is responsible for the cytotoxic activity [64]. 

A preclinical study showed that the PMs accumulated in solid tumors in mice by the EPR effect and DOX released from the inner core by diffusion exerted significantly higher antitumor activity than free DOX [155]. Concerning the release of DOX from the conjugated block copolymer, administration of a DOX-conjugated polymer to dogs showed that DOX concentration in plasma was 100-fold less than that of NK911 containing the same amount of DOX-conjugated polymer. Thus, conjugated DOX does not influence plasma DOX concentrations after IV injection of NK911 [64].

NK911 was the first polymeric micellar formulation to proceed into clinical trials, in 2001. In a phase 1 clinical trial enrolling 23 patients with metastatic or recurrent solid tumors refractory to conventional chemotherapy, IV administration of NK911 (6–67 mg/m^2^) every 3 weeks showed that the formulation was well tolerated, without any infusion-related reactions [64]. A partial response was obtained in one patient with metastatic pancreatic cancer and eight patients exhibited stable disease for longer than 4 weeks. Neutropenia was the main hematological toxicity, with DLTs observed at a dose of 67 mg/m^2^ (grade 4 neutropenia lasting more than 5 days), which was determined as the MTD. Common non-hematological toxicities included mild alopecia, stomatitis, and anorexia. The recommended phase 2 dose was established as 50 mg/m^2^ every 3 weeks [64]. At this dose level, the plasma AUC of NK911 was 2-fold higher than that of free DOX but more than 100-fold lower than that of Doxil^®^ (PEGylated liposomes), indicating that NK911 is less stable in plasma than the liposomal formulation. However, the volume of distribution at a steady state of NK911 was nearly 180-fold higher than that of Doxil^®^ at the same dose level, suggesting that the distribution of DOX in tumor tissue may be wider in the case of the PMs when compared to the nanoliposomes upon extravasation from the tumor vessels [64].

#### 4.3.2. SP1049C

SP1049C, developed by Supratek Pharma Inc. (Montreal, QC, Canada), is a P-gp-targeting polymeric micellar formulation of DOX, which is non-covalently incorporated in mixed micelles (22–27 nm) made from a blend of triblock copolymers, Pluronic^®^ L61 and Pluronic^®^ F127 (1:8 *w*/*w*) [83,156,157]. Pluronic^®^ L61 was shown to sensitize DOX-resistant cancer cells through an interplay between ATP depletion, membrane fluidization, and inhibition of P-gp ATPase activity, while Pluronic^®^ F127 provided micellar stabilization [156,157]. SP1049C was effective in vitro against MDR cells normally not susceptible to DOX, which was attributed to an increase in drug uptake, energy-dependent drug efflux inhibition, and changes in intracellular drug trafficking [64]. SP1049C also exhibited improved antitumor efficacy in vivo against drug-resistant tumors due to enhanced tumor accumulation through the EPR effect while distribution in liver, kidney, heart, and lung was similar to conventional DOX but with higher brain levels for SP1049C [156]. 

A phase 1 dose-escalation trial in patients with advanced cancer (n = 26) administered SP1049C (5–90 mg/m^2^) as an IV infusion once every 3 weeks for up to six cycles showed myelosuppression as a DLT at 90 mg/m^2^ [66]. The MTD was 70 mg/m^2^ and was also the RD for phase 2 studies [66]. The pharmacokinetic profile of SP1049C showed a slower clearance when compared with conventional DOX [66]. Antitumor activity following SP1049C administration was observed in three patients with advanced resistant solid tumors (Ewing’s sarcoma, carcinosarcoma, and esophageal adenocarcinoma) that had received prior therapy [66]. Nausea, vomiting, fatigue, and alopecia were common side effects but palmar plantar erythrodysthesia was not observed [66].

A subsequent phase 2 study in 21 patients with metastatic or locally advanced unresectable adenocarcinoma of the esophagus or gastroesophageal junction (chemotherapy naïve) treated with SP1049C (75 mg/m^2^, IV infusion) every 3 weeks showed an ORR of 47%, median OS of 10 months, and PFS of 6.6 months. Grade 3/4 neutropenia (61.9%) was the main drug-related AE [158]. Non-hematological AEs included alopecia (66.7%), mucositis (47.6%), anorexia (19%), vomiting (19%), nausea (14.3%), and lethargy (14.3%) [158]. Asymptomatic and small decrements (grade 1) in left ventricular ejection fraction were observed in four patients, which discontinued treatment [158]. A phase 3 clinical study of SP1049C in metastatic adenocarcinoma of the upper gastrointestinal tract was started but no results have been reported, although SP1049C was granted orphan drug status by the FDA for esophageal carcinoma in 2005 and for gastric cancer in 2008 [83,159]. 

#### 4.3.3. NC-6300

NC-6300, developed by NanoCarrier Co., Ltd. (Chiba, Japan), is a pH-sensitive polymeric micellar formulation with a particle size of 40–80 nm composed of PEG_12k_ and poly(α,β-aspartic acid) block copolymers, the latter partially modified with benzyl groups for stabilization of the micellar structure, and conjugated with EPI via an acid-labile hydrazone bond [160]. 

NC-6300 accumulates in tumor tissue due to the EPR effect and selectively releases the drug in the acidic TME [160]. In vitro drug release studies showed that at pH 3.0, 80% of EPI was released from NC-6300 within 1 h while at pH 7.0 or 7.4 only 20% of the drug was released within 24 h [160]. Pharmacokinetic studies in rats showed highly enhanced plasma retention of NC-6300 compared with native EPI (AUC 116.7 versus 0.053 μg·h/mL, respectively, both intravenously administered at 1 mg/kg dose) [160]. NC-6300 (15 and 20 mg/kg three times with a 4-day interval between doses) was able to regress a Hep3B human hepatic tumor and to inhibit the growth of MDA-MB-231 human breast tumor in xenografted mice while EPI (7 mg/kg at the same schedule) only slowed tumor growth [160]). Tissue distribution studies of NC-6300 (20 mg/kg) showed efficient release of EPI in the tumor, with a release ratio of 74% against 20–46% in healthy tissues. The AUC value of released EPI in the tumor and in the heart was 4.3-fold higher and 0.28-fold lower, respectively, compared with the native free EPI solution at the same dose, resulting in a 15-fold higher therapeutic index for the polymeric micellar formulation [160].

Preclinical evaluation of NC-6300 (10 or 15 mg/kg weekly, for 3 weeks) in mice bearing subcutaneous or orthotopic xenografts of human hepatocellular carcinoma Hep3B cells showed that the formulation improved drug antitumoral activity and survival rate when compared with conventional EPI (10 mg/kg, at the same schedule), with no significant cardiotoxic effects [161]. NC-6300 increased EPI concentrations in the plasma, liver, spleen, and tumor and decreased drug levels in the kidney, lung, and heart compared with the native drug [161].

NC-6300 has also been conjugated with an antitissue factor mAb (clone 1849) for targeted cancer therapy, since tissue factor (TF), an initiator of the extrinsic blood coagulation cascade, is frequently overexpressed in cancer cells and tumor vascular endothelium [162]. The antitumoral activity of anti-TF-NC-6300 was higher in mice bearing tumor xenografts with high TF expression (human gastric cancer 44As3 cells and human pancreatic cancer BxPC3 cells) compared with NC-6300 but in low-TF-expressing xenografts (human pancreatic cancer SUIT2 cells) both formulations showed similar activity, although with higher tumor accumulation of anti-TF-NC-6300. 

However, clone 1849 antibody was found to inhibit TF-associated blood coagulation activity and was replaced by clone 1859 in a subsequent study, which had no effect on blood coagulation [163]. The novel anti-TF-NC-6300 formulation showed higher in vitro cytocidal effects in BxPC3 cells compared with NC-6300 but not in the SUIT2 cell line. Similarly, the in vivo tumor growth inhibition efficacy of anti-TF-NC-6300 was superior to NC-6300 in BxPC3 xenografts, but not in the SUIT2 xenograft model, demonstrating the enhanced antitumor effect of anti-TF-NC-6300 in the high-TF-expressing tumor [163]. 

Targeted immunotherapy with NC-6300 in combination with anti-PD-L1 antibody was found to potentiate immune checkpoint inhibition in mouse models of osteosarcoma and fibrosarcoma, and NC-6300 was even more effective than the MTD of DOX at increasing tumor growth delay induced by anti-PD-L1 antibody [164]. Further mechanistic studies showed that NC-6300 induced immunogenic cell death and normalized the TME, and the combination with anti-PD-L1 antibody increased the intratumoral density and proliferation of T cells [164].

The mechanisms underlying the antitumoral effects resulting from the combination of NC-6300 and high-intensity focused ultrasound (HIFU) were investigated in human pancreatic adenocarcinoma (BxPC-3) and human promyelocytic leukemia (HL-60) cell lines [165]. The sonodynamic therapy (SDT) system employed a specific HIFU irradiation sequence consisting of a short-duration high-intensity triggering pulse (2000 W/cm^2^, 0.02 ms) to generate cavitation bubbles and a heating wave (10–1000 W/cm^2^, 10 ms) for sustention of cavitation bubbles and bubble-enhanced heating [165]. The combination of NC-6300 with trigger-pulsed HIFU (TP-HIFU) was shown to increase ROS production in vitro without drug degradation due to the protective hydrophilic shell of the micelles [165]. Moreover, the generation of superoxide anions by TP-HIFU increased upon the addition of NC-6300, and the sonosensitizer potency of NC-6300 was superior to that of EPI.

SDT based on the combination of a low dose of NC-6300 (2.5 mg/kg, 24 h prior to HIFU irradiation) and low-energy HIFU (270 or 360 W/cm^2^) showed improved efficacy in mouse models of colon cancer (Colon-26) and pancreatic cancer (MIA PaCa-2) compared with NC-6300 monotherapy or HIFU alone [166]. SDT with NC-6300 (7.5–30 mg/m^2^) and HIFU (9–30 sequences) were effective in the treatment of canine cancer in four pet dogs with spontaneous tumors (chondrosarcoma, osteosarcoma, hepatocellular cancer, and prostate cancer) with no AEs after five SDT sessions [167].

A first-in-human phase 1, dose-escalation study of NC-6300 in patients with advanced or recurrent solid tumors (n = 19) administered NC-6300 (15–225 mg/m^2^) as 10 min IV infusion every 3 weeks showed a partial response in one patient with breast cancer and stable disease in ten patients [168]. The recommended phase 2 dose was set as 170 mg/m^2^, which was also the MTD. The human pharmacokinetic profile of NC-6300 was linear and consistent with preclinical studies in rats and monkeys. 

A phase 1b, dose-escalation trial of NC-6300 monotherapy (125–215 mg/m^2^, IV, every 3 weeks) in patients (n = 29) with advanced, metastatic, or unresectable solid tumors, including soft-tissue sarcomas (n = 11), showed an ORR of 11%, with partial responses in angiosarcoma and endometrial stromal sarcoma. DLTs included grade 3/4 neutropenia, thrombocytopenia, anemia, febrile neutropenia, stomatitis, and lung infection [67]. The MTD and RD for phase 2 studies were determined to be 185 mg/m^2^ and 150 mg/m^2^, respectively. Based on the promising antitumor activity against angiosarcoma, an expansion cohort was undertaken (NCT03168061) which enrolled 10 patients [169]. Administration of NC-6300 at the RD (150 mg/m^2^, IV), once every 3 weeks, resulted in a median PFS of 5.4 months (3.8 months and 8.2 months in patients with and without prior anthracycline treatment, respectively) [169]. The most common AEs were neutropenia, thrombocytopenia, leukopenia, anemia, fatigue, and nausea [169].

### 4.4. Platinum-Based Micelle Formulations

Cisplatin (CDDP) is a platinum coordination complex with a broad spectrum of antitumor activity used as first-line therapy for several solid tumors, including breast, ovarian, testicular, bladder, head and neck, liver, and small-cell and non-small-cell lung cancers, either alone or in combination with radiation and/or other chemotherapeutic drugs [170,171].

The potent genotoxicity of cisplatin, activated intracellularly by the aquation of the two chloride leaving groups, results from the formation of mainly intrastrand but also interstrand DNA crosslinks through coordination bonds between the platinum atoms and the purine nucleobases [170,171]. However, non-selective distribution of the drug results in acute dose-related severe AEs, namely nephrotoxicity, myelosuppression, neurotoxicity, and ototoxicity, and the therapeutic effect is further limited by intrinsic or acquired drug resistance [171]. 

Platinum complexes like cisplatin undergo stepwise aquation reactions in which the chloride ions are replaced by water molecules leading to the pharmacologically active cationic mono- and diaqua complexes [170,171]. The rates of aquation of platinum complexes depend on the concentration of chloride ions in the media, thus the drugs are relatively stable in plasma due to the high chloride concentration (100 mM). On the other hand, the low intracellular chloride concentration (4–12 mM) promotes the formation of the cationic aqua forms of the platinum complexes, which do not readily diffuse out of the cell since their charge hinders crossing of the lipophilic cellular membrane, becoming trapped within the cell and binding to intracellular targets, mainly DNA but also RNA and proteins [170].

Carboplatin is a second-generation platinum designed to reduce cisplatin DLT by replacing the readily exchangeable chloride ligands with a bidentate 1,1-cyclobutanedicarboxylic acid ligand, thus slowing down the rate of aquation reactions, which also reduces the drug potency [170,171]. However, the lower excretion rate of carboplatin results in higher retention (half-life of 30 h versus 1.5–3.6 h for cisplatin) and longer-lasting effects [170]. Carboplatin has reduced nephrotoxic side effects compared with cisplatin but is associated with severe myelosuppression. 

Oxaliplatin is a third-generation platinum complex developed to overcome cellular resistance to cisplatin and carboplatin. The parent compound, cis-dichloro(1,2-diamminocyclohexane)platinum(II) (DACHPt), is a potent anticancer agent with a broader spectrum of activity and no crossresistance with cisplatin and carboplatin, obtained by replacing the two amine groups of cisplatin by 1,2-diamminocyclohexane (DACH) [171]. However, DACHPt lacks water solubility (0.25 mg/mL compared with 1.2 mg/mL for cisplatin), which was enhanced in oxaliplatin by replacing both chloride ligands with a bidentate oxalate ligand [170]. Oxaliplatin undergoes rapid non-enzymatic biotransformation due to displacement of the labile oxalate group by water and nucleophiles, namely chloride ions, present in biological media to form cytotoxic mono- and diaqua/chloro platinum complexes, which complicates the drug pharmacokinetics. The higher cytotoxic activity of oxaliplatin compared with cisplatin and carboplatin has been attributed to the bulky DACH ligand, which induces DNA lesions that are poorly recognized and/or prevent binding by DNA repair enzymes [170]. Moreover, oxaliplatin can induce ribosome biogenesis stress and enable immunogenic cell death by promoting a T-cell-dependent immune response, and these differing modes of action further contribute to the efficacy of oxaliplatin in cisplatin-resistant cell lines [171].

Oxaliplatin (Eloxatin^®^, Sanofi-Aventis) is indicated as the first-line treatment of metastatic CRC, in combination with 5-fluorouracil and leuvocorin, known as the FOLFOX regimen, and as adjunctive therapy after resection of the primary tumor. Oxaliplatin lacks the nephrotoxicity of cisplatin and the severe myelosuppression of carboplatin but induces severe peripheral neuropathy, often the DLT, characterized by acute neuropathy that includes acral paresthesia and dysesthesia triggered or enhanced by exposure to cold, and chronic neuropathy, with loss of sensory and motor function after long-term treatment [170,171]. 

#### 4.4.1. NC-6004 (Nanoplatin™)

NC-6004 (Nanoplatin™), developed by NanoCarrier Co., Ltd. (Chiba, Japan) and licensed to Orient Europharma Co., Ltd. (Taiwan), is a cisplatin polymeric micellar formulation comprising amphiphilic diblock copolymers made of PEG and p(Glu) with a micelle size around 30 nm prepared through polymer–metal complex formation between cisplatin and the carboxylate groups of the hydrophobic segment [83,172]. In physiological media, NC-6004 is expected to release cisplatin through an exchange reaction between the carboxylate groups of p(Glu) and chloride ions present in the media [172].

NC-6004 was stable in distilled water in long-time storage but in physiological saline a sustained drug release was observed, lasting longer than 150 h, which was accompanied by the decay of the carrier micelles [172]. The micelles had remarkably prolonged blood circulation and showed high selectivity towards cancer cells in Lewis-lung-carcinoma-bearing mice, effectively accumulating in solid tumors by passive targeting through the EPR effect and showing reduced accumulation in normal tissues (kidney, liver, spleen, and muscle) [172]. Both NC-6004 and free cisplatin, at the same dose regimen (4 mg/kg, five 2-day cycles), had significant antitumor activity in murine colon adenocarcinoma 26 (C 26)-bearing mice compared with a non-treated control group, but only NC-6004-treated mice showed complete tumor regression without significant body weight loss [172]. 

Further studies showed that the secondary structure in the polypeptide segment of the PEG-*b*-p(Glu) copolymer, characterized by α-helix bundles in the micellar core, was critical for stabilization of micellar structure against dilution in physiological media, prolonging blood circulation and achieving sustained drug release in the TME through surface erosion of the bundled core by chloride ions and disassembly of the micelles [173]. The formulation accomplished selective tumor accumulation after IV injection (4 mg/kg, three times at 2-day interval) in mice bearing subcutaneous human pancreatic adenocarcinoma (BxPC3) xenografts, with reduced non-specific distribution to the liver and spleen, and effectively suppressed tumor growth [173].

The in vitro growth inhibitory effect of NC-6004 against human oral squamous cell carcinoma cell lines (OSC-19, OSC-20, HSC-3, and HSC-4) was inferior to that of cisplatin [174]. However, both formulations showed comparable in vivo antitumor effects in an orthotopic tongue cancer (OSC-19) mouse model, with the micellar formulation providing a better safety profile, with minimal renal cell damage [174]. Moreover, NC-6004 showed prolonged blood and lymphatic circulation, reducing the incidence of lymphatic metastasis compared with cisplatin [174].

The antitumor effect of NC-6004 (0.5, 2.5, or 5.0 mg/kg IV, every 3 days, for a total of three injections) in nude mice implanted with the human gastric cancer cell line MKN-45 was comparable to or higher than that of cisplatin at the same dose schedule, with tumor concentrations of platinum peaking at 10 min and 48 h after administration of cisplatin and NC-6004, respectively [175]. Additionally, combined treatment with NC-6004 and S-1 in mice bearing human gastric cancer (44As3Luc) xenografts showed significantly lower body weight loss compared with cisplatin plus S-1 while retaining similar antitumor activity.

In healthy rats, a single IV injection of NC-6004 significantly inhibited the nephrotoxicity and neurotoxicity of cisplatin, according to data from histopathological and biochemical studies [175]. Renal platinum concentrations at 10 min and 1 h after administration of NC-6004 (5 mg/kg) were 11.6- and 3.1-fold lower, respectively, compared with the cisplatin (5 mg/kg) group [175]. Rats treated with cisplatin (10 mg/kg) also showed higher plasma concentrations of blood urea nitrogen (BUN) and creatinine compared with animals given NC-6004 (10 and 15 mg/kg). Thus, NC-6004 may facilitate treatment on an outpatient basis due to decreased renal toxicity since it does not require hospitalization for hydration to prevent cisplatin nephrotoxicity [175,176]. The neurophysiological examination was performed after 11 IV administrations of NC-6004 or cisplatin, both at 2 mg/kg, twice a week [175]. Contrary to NC-6004-treated rats, the cisplatin-treated rats showed a significant delay in sensory nerve conduction velocity in their hind paws and degeneration of the sciatic nerve, which was attributed to significantly reduced accumulation of platinum in nerve tissue following NC-6004 administration [175]. 

NC-6004 also prevented ototoxicity, a common side effect of high-dose cisplatin therapy, in healthy guinea pigs. Treatment with NC-6004 (8 or 12 mg/kg bolus IV injection) resulted in no apparent auditory brainstem responses (ABRs) while treatment with cisplatin (8 or 12 mg/kg bolus IV injection plus subcutaneous hydration with 20 mL normal saline to avoid renal damage) lead to dose-dependent threshold shifts and significant hair-cell loss [177]. Platinum distribution and concentration in the organ of Corti were significantly reduced in NC-6004-treated guinea pigs compared with the cisplatin-treated group, indicating that NC-6004 prevented cisplatin-induced ototoxicity by circumventing the vulnerable hair cells in the inner ear due to the micelle’s size (around 30 nm), which does not allow crossing the blood–cochlear barrier through the intrastrial space (around 15 nm) [177].

The first-in-human phase 1, dose-escalation study of NC-6004 (10–120 mg/m^2^, IV, every 3 weeks) in patients with solid tumors (n = 17) conducted in the UK revealed renal impairment and hypersensitivity reactions at a dose of 120 mg/m^2^, despite implementation of a premedication regimen and posthydration, which was established as the MTD [69]. Only one DLT occurred in a patient treated with 90 mg/m^2^ of NC-6004, which was determined as the RD. Seven patients showed stable disease. Pharmacokinetic analysis showed that IV administration of NC-6004 reduced *C*_max_ and increased AUC compared with cisplatin at the same dose (120 mg/m^2^), indicating that the delayed and sustained release of cisplatin following IV administration of NC-6004 contributed to its lower toxicity and better safety profile compared to conventional cisplatin [69]. 

Results from a phase 1/2 study (NCT00910741) conducted in Asia (Taiwan and Singapore) enrolling patients with pancreatic cancer (n = 19) treated with NC-6004 (30, 60, 90, and 120 mg/m^2^ every 3 weeks on day 1) plus gemcitabine (1000 mg/m^2^ twice every 3 weeks, on days 1 and 8) registered two DLTs at 120 mg/m^2^, which was determined as the MTD, while the RD was established as 90 mg/m^2^. These values were identical to the ones obtained for the monotherapy regimen in the UK study [178]. However, the combination regimen showed modest efficacy, with one partial response and ten patients experiencing stable disease among the seventeen in the evaluable population. A prophylactic two-dose oral steroid regimen was implemented to reduce the risk of hypersensitivity reactions, which were not observed [178]. Nevertheless, NC-6004 entered and completed a phase 3 clinical trial (NCT02043288) for evaluation of the impact of NC-6004 addition to gemcitabine in the treatment of locally advanced or metastatic pancreatic cancer in Asian patients, but no results have been posted yet. 

An open-label phase 1 study of NC-6004 (60 or 90 mg/m^2^, IV, on day 1, in every 3-week cycle) in combination with gemcitabine (1000 mg/m^2^, IV, on days 1 and 8 starting on the second cycle) in Japanese patients with advanced solid tumors (n = 12) established both the MTD and the RD at 90 mg/m^2^ [179]. One patient showed partial response while eight patients had stable disease. The most frequent drug-related AEs were neutrophil decrease (66.7%) and WBC count decrease (41.7%). Prophylactic hydration therapy before and after treatment with NC-6004 was necessary to prevent renal toxicity [179]. 

In a phase 1b/2 study (NCT02240238) conducted in the US and Europe, patients with advanced solid tumors (n = 22) were treated with NC-6004 (60–180 mg/m^2^ on day 1) and gemcitabine (1250 mg/m^2^ on days 1 and 8) every 3 weeks [180]. The most common grade 3/4 hematologic AEs were leukopenia (68%) and thrombocytopenia (59%), and the MTD and phase 2 RD were both determined to be 135 mg/m^2^, higher than the corresponding values reported in the UK study [69] and the Asian study [178]. The inconsistency may be due to different study designs since the latter studies used a 3 + 3 modified Fibonacci dose escalation design while the US study adopted the Bayesian continual reassessment model. No clinically significant nephro-, neuro-, or ototoxicity was observed. Among 20 evaluable patients, 3 showed partial responses (15%), 14 had stable disease (70%), and tumor shrinkage was detected in 11 patients (55%) [180]. 

The expansion phase 2 study (NCT02240238) of NC-6004 (135 mg/m^2^ on day 1) plus gemcitabine (1250 mg/m^2^ on days 1 and 8) in patients with squamous NSCLC (n = 34), biliary tract cancer (n = 50), or bladder cancer (n = 13) showed a median PFS of 3.9, 4.3, and 6.8 months, respectively [68]. The most common grade 3 treatment-emergent AEs were nausea, anemia, neutropenia, and hyponatremia, independently of the type of cancer [68]. 

A phase 2a/2b clinical trial (NCT03771820) of NC-6004 plus the checkpoint inhibitor pembrolizumab in patients with HNSCC refractory to platinum or platinum-containing regimens (n = 16) treated with the recommended phase 2 dose (135 mg/m^2^) demonstrated that the formulation was safe and well tolerated [181]. No grade 3/4 toxicity or clinically significant nephro-, neuro-, or ototoxicity was observed, despite the RD being higher than conventional cisplatin therapeutic doses, and the most common side effect was hypomagnesemia (31%). Three patients showed partial response (21%) while tumor shrinkage occurred in eight patients (57%) [181]. 

Based on the promising therapeutic efficacy and safety of the NC-6004 cisplatin formulation, NC-4016 was developed for the delivery of oxaliplatin in order to modify its pharmacokinetics, improve antitumor activity, and decrease drug-related toxicity, as discussed in the next section.

#### 4.4.2. NC-4016

NC-4016, developed by NanoCarrier Co., Ltd. (Chiba, Japan), is a polymeric micellar formulation of DACHPt, the oxaliplatin parent complex, composed of PEG_12k_-p(Glu)_6k_ diblock copolymers [182,183]. The PMs, with 30–40 nm size, were prepared through polymer–metal complexation between DACHPt and the carboxylic groups of the hydrophobic copolymer [182,183]. The platinum complexes are released from the micelle core by exchange reaction between chloride ions present in the media and carboxylic groups of the p(Glu) copolymer complexed with DACHPt. Drug release studies performed in PBS (pH 7.4) at 37 °C, mimicking physiological conditions, demonstrated the in vitro sustained release of platinum from NC-4016 after an induction period of 12 h [183]. 

The growth inhibitory effect of NC-4016 in a human cervical adenocarcinoma (KB) cell line was weaker than that of oxaliplatin in vitro, presumably due to the slow-release behavior of DACHPt complexes from the micelles [184]. However, the in vivo antitumoral efficacy of the micellar formulation was superior to that of oxaliplatin at equivalent Pt doses in nude mice bearing subcutaneous KB xenografts, which was attributed to higher Pt concentrations in tumor [184]. A 74% reduction in tumor weight was achieved after administration of NC-4016 compared with only 33% in the oxaliplatin-treated group [184]. Additionally, in a rat model of oxaliplatin-induced neuropathic pain, no acute cold hypersensitivity was observed in the NC-4016-treated group, contrary to the oxaliplatin group [184]

NC-4016 was shown to maintain micelle form during blood circulation, extravasating from blood vessels into tumor tissues and selectively dissociating within late endosomes, thus enhancing drug delivery to the nucleus of cancer cells compared with free oxaliplatin [185]. The high intratumoral accumulation of NC-4016 micelles, combined with their subcellular drug targeting, avoiding cytoplasmic detoxification systems and improving intracellular delivery, allowed the formulation to overcome oxaliplatin resistance in vivo in a human CRC model (HT29 and HT29/ox xenografts) [185].

In a mouse model of multiple liver metastases from murine colon adenocarcinoma (C26 cells), mice treated with NC-4016 showed significant reduction in metastatic nodules and liver weight compared with mice treated with oxaliplatin [186]. High levels of NC-4016 were found to accumulate in metastatic livers, producing a strong antitumor effect without severe AEs, which was attributed to significantly higher accumulation of the micellar formulation in the metastatic liver compared with oxaliplatin [186]. On the other hand, distribution of the PMs in healthy livers was limited, demonstrating their selectivity for tumor tissue [186]. 

The NC-4016 micelles, due to their small size, were able to penetrate poorly permeable pancreatic tumors in mice [187] and to accumulate in orthotopic scirrhous gastric tumors, inhibiting tumor growth and their lymph node metastasis [188]. Repeated systemic administration of NC-4016 (2 mg/kg weekly, for 8 weeks) in a transgenic mouse model of spontaneous murine pancreatic cancer inhibited the growth of primary tumors due to efficient accumulation and penetration in tumor tissue and reduced the development of metastases and ascites, preventing peritoneal metastasis and prolonging the survival of mice [189]. The model used elastase-1-promoted luciferase and simian virus 40 T and t antigens (EL1-luc/TAg) transgenic mice, which continuously express SV40 T oncogene, and can gradually develop cancer and metastasis under viable immunity, angiogenesis, and inflammation processes, consistent with the evolution of human pancreatic cancer, thus avoiding altered microenvironment features present in allograft and xenograft tumor models that may affect the behavior of nanocarriers [189].

Combination therapy with NC-4016 and NC-6300 (EPI micelles) provided higher synergistic activity in mice bearing human gastric (44As3Luc cells) xenografts, exhibiting higher antitumoral activity against the subcutaneous xenografts and improving OS in the orthotopic tumor model compared with the combination EPI plus oxaliplatin, with lower cardiotoxicity and neurotoxicity [190]. The intratumoral concentrations of EPI and platinum were significantly higher upon administration of the PMs in comparison with the conventional drug combination [190].

Functionalization of NC-4016 with the cRGD peptide has been performed based on the peptide inhibitory activity against the development of metastasis and the cytotoxic activity of the DACHPt-loaded PMs, and the capability of cRGD-installed NC-4016 PMs for cooperatively inhibiting the formation and progression of lymph node metastasis was assessed in a syngeneic melanoma model [191]. Both conjugated and non-conjugated micelles showed comparable antitumor activity against the primary tumors and the established metastatic foci in lymph nodes [191]. On the other hand, the conjugated micelles significantly enhanced the efficacy against lymph node metastasis draining from primary tumors through improved inhibition of melanoma cell migration due to the synergistic effect [191].

Additional loading of NC-4016 PMs with gadolinium-diethylenetriaminpentaacetic acid (Gd-DTPA), a magnetic resonance imaging contrast agent, was performed to obtain a formulation suitable for simultaneous imaging and therapy in an orthotopic rat model of hepatocellular carcinoma (N1-S1 rat hepatoma cells) [192]. The incorporation of drug and contrast agent in the micelles corresponded to 45% and 5% of the carboxylic groups in PEG-*b*-p(Glu) copolymers, respectively, and the double-loaded nanocarrier had a size of 33 nm. After a single injection of Gd-DTPA/DACHPt-loaded micelles into the hepatic artery, the micelles achieved strong and specific tumor contrast enhancement, induced high levels of tumor apoptosis, and significantly suppressed tumor size and growth without severe AEs [192]. Survival rate was significantly improved compared with oxaliplatin and saline control groups [192]. 

A phase 1 dose-escalation and pharmacokinetic study (NCT03168035) of NC-4016 (15–80 mg/m^2^ IV infusion over 2 h, every 3 weeks) in 34 patients with advanced solid tumors or lymphoma was completed in 2017 but no results have been published yet.

## 5. Polymeric Micelles for Cancer Immunotherapy

Cancer immunotherapies, involving stimulation of innate and adaptive immune responses crucial to antitumor immunity, are mainly based on immune checkpoint inhibitors (ICIs), such as antiprogrammed cell death protein 1 (PD-1) and antiprogrammed death-ligand 1 (PD-L1) antibodies, cell-based therapies like engineered chimeric antigen receptor T cells (CAR-T), and cancer vaccines [193]. However, in many solid tumors the success of immunotherapy is often hampered by primary and acquired resistance. NP-based approaches to improve immune cell infiltration into the immunosuppressive TME have the potential to enhance drug efficacy and overcome therapy resistance [193]. 

The cytosolic DNA sensing cyclic GMP-AMP synthase (cGAS)-stimulator of interferon genes (STING) pathway represents a promising target for cancer immunotherapy. STING is endogenously activated by 2′,3′-cyclic GMP-AMP (cGAMP), a cyclic dinucleotide synthesized by cGAS in response to cytosolic DNA as a danger signal. Acute genomic stressors induced by radiation, cisplatin, and intrinsic DNA damage are known to generate cytosolic DNA responsible for cGAS-STING activation in cancer cells [194]. Activation of STING by endogenous cGAMP or cGAMP agonists mediates a type-I interferon (IFN-I) response that promotes the maturation and migration of dendritic cells, which in turn presents tumor-associated antigens on major histocompatibility complexes to activate CD8^+^ T cells for tumor-specific cell killing [194,195].

### ONM-501

ONM-501, developed by OncoNano Medicine (TX, Southlake, USA), is a dual-activating polyvalent STING agonist comprising cGAMP loaded into PMs made from a STING-activating, pH-sensitive diblock copolymer, (PEG)_114_-*b*-poly [2-(hexamethyleneimino)ethyl methacrylate]_90_ (PC7A), based on OncoNano OMNI™ proprietary polymer technology, part of the company’s proprietary pH-activated micelle platform (ON-BOARD™).

The ultrapH-sensitive (UPS) PC7A copolymers (p*K*_a_ 6.9), comprising ionizable seven-membered ring heterocyclic tertiary amine side chains in the hydrophobic segment, exhibit a highly cooperative protonation process upon pH-triggered self-assembly at a critical micellization protonation degree (CMPD) of 0.85 [196]. Below the pH transition threshold (pH < 6.9), the tertiary amine groups become highly protonated, and the micelles dissociate into water-soluble cationic unimers (hydrodynamic diameters < 10 nm) while above the threshold (pH > 6.9) the unimers are deprotonated and become hydrophobic, driving the formation of core–shell PMs (hydrodynamic diameters around 30 nm) [196]. The protonation cooperativity driven by the phase transition of PC7A copolymers occurs at a sharp pH and turns the copolymers into pH-triggered “on–off” switchable nanocarriers due to the bimodal proton distribution between highly protonated unimer (“on”) and neutral micelle (“off”) states [196]. Thus, PC7A micelles are stable at physiological pH of 7.4, preventing drug leakage and prolonging blood circulation while achieving instantaneous payload release upon exposure to acidic microenvironments [196]. PC7A micelles have been used in tumor vaccines to target early endosomal pH (6.5–7.0) for enhanced cytosolic delivery of tumor antigens and crosspresentation to antigen-presenting cells (APCs) in draining lymph nodes [197].

Furthermore, the ability of PC7A copolymers to bind and activate STING through polyvalent phase condensation led to elevated expressions of costimulatory molecules (CD86) on dendritic cells and rapid release of type-I IFNs, boosting antitumor immunity for cancer immunotherapy [197]. Thus, PC7A is a potent vaccine adjuvant, and PC7A nanovaccines (0.5 μg antigen, 30 μg polymer) showed robust cytotoxic T cell response with low systemic cytokine expression, significantly inhibiting tumor growth in multiple tumor mouse models, including B16-F10 melanoma, colon cancer MC38, and human papillomavirus (HPV)-E6/E7 TC-1 tumors upon subcutaneous injection 5 days after tumor inoculation, followed by a booster shot 5 days later [197]. Combination of the STING-activating PC7A nanovaccine with anti-PD-1 mAb, an immune checkpoint inhibitor, displayed a synergistic effect with 100% survival over 60 days in a TC-1 tumor model. Rechallenging of the tumor-free animals with TC-1 cells led to complete inhibition of tumor growth, suggesting that nanovaccine-induced activation of memory T cells is responsible for the generation of a long-term antitumor response [197]. Interestingly, intratumoral delivery of PC7A nanovaccine achieved stronger antitumor immunity and efficacy compared with subcutaneous delivery [198].

Further studies demonstrated the polyvalent PC7A-induced phase condensation mechanism for STING activation and revealed that PC7A binds to a non-competitive surface-binding site on the protein distinct from the cGAMP-binding pocket (or other cyclic dinucleotides), resulting in prolonged expression of interferon-stimulated genes (*Ifnb1* and *Cxcl10*) compared with the endogenous ligand, with PC7A retaining immune activity in several cGAMP-resistant STING variants [199]. Moreover, cGAMP-loaded PC7A micelles (2.5 μg/50 μg polymer), i.e., ONM-501, showed a synergistic antitumor immune response in tumor-bearing mice, significantly improving long-term survival compared with either free cGAMP (2.5 μg) or empty PC7A micelles (50 μg), upon intratumoral injection [199], as well as in vitro synergistic STING activation in resected human tumors and lymph nodes [199].

At physiological pH, ONM-501 micelles protect cGAMP from enzymatic degradation, prevent drug leakage and systemic toxicity, and enable targeted endolysosome delivery in the acidic pH of the TME through micelle dissociation, releasing both the endogenous ligand and the STING-activating polymer, which produces dual “burst” and “sustained” STING activation, observed across different species [195,200]. Increased IFNB1 and CXCL10 mRNA levels have been found in peripheral blood mononuclear cells (PBMCs) of rats, mice, beagle dogs, cynomolgus monkeys, and humans after 24 h treatment with ONM-501, consistent with STING activation [200].

Single- and multiple-dose toxicology studies in healthy rats and cynomolgus monkeys showed that ONM-501 (7.5–30 mg/kg, subcutaneously) was well tolerated without severe systemic AEs [201]. Dose-dependent increases in lymphocytes and cytokines were observed, consistent with STING activation [202]. Pharmacokinetic and biodistribution studies (in vivo and ex vivo) revealed that systemic exposure of mice to ONM-501 (2.5 μg/50 μg polymer) was lower after intratumoral administration than after subcutaneous administration to healthy mice, consistent with increased retention of both active moieties of ONM-501 (cGAMP and PC7A) within tumors [202].

The combination of cGAMP canonical binding and PC7A polymer non-canonical binding results in synergistic STING activation and provides potent antitumor efficacy in multiple murine syngeneic tumor models, which was further enhanced by combination with the anti-PD1 checkpoint inhibitor [201]. The combination regimen improved therapeutic outcomes compared with monotherapies, in both anti-PD1-sensitive (“hot”) and -resistant (“cold”) models, and prolonged long-term survival over 100 days in 30%, 40%, 50%, and 80% of animals in MC38, CT26, B16-F10, and A20 tumor models, respectively. Pharmacodynamic analysis showed enhanced tumor T cell infiltration and ONM-501-upregulated PD-L1 expression in tumor tissue [201]. Moreover, tumor inhibition was observed in both primary and distal MC38 tumors in the same animal after treatment with ONM-501 (three intratumoral injections with 3-day interval), the systemic abscopal effect being confirmed by the reduction of lung metastasis in an immune “cold” triple-negative orthotopic breast cancer 4T1 model [200]. ONM-501 has recently entered a first-in-human multicenter phase 1, dose-escalation and dose-expansion study (NCT06022029) in patients with advanced solid tumors and lymphomas, as intratumorally delivered monotherapy and in combination with intravenous cemiplimab-rwlc (Libtayo^®^, Regeneron), an anti-PD1 mAb [196].

## 6. Polymeric Micelles for Cancer Diagnosis

Polymeric micellar formulations offer several advantages compared with small-molecule contrast agents, including long blood-pool residence times, tumor accumulation by the EPR effect, potential for cancer active targeting and triggered release of payloads, and tunable biodistribution for enhanced contrast specificity between tumor and healthy tissues [28].

In cancer imaging-based diagnosis and monitoring, X-ray computed tomography (CT), single-photon emission computed tomography (SPECT), magnetic resonance imaging (MRI), positron emission tomography (PET), optical imaging, and ultrasonography play important roles [22]. Tumor, node, metastasis (TNM) staging, objective response, and left ventricular ejection fraction (LVEF) are relevant imaging biomarkers in clinical oncology [22].

Moreover, these non-invasive and real-time imaging technologies provide valuable information regarding nanomedicines’ pharmacokinetics and biodistribution, tumor accumulation and penetration, and drug release profile, once the appropriate formulation for incorporation of imaging agents and the suitable imaging modality are chosen [28]. In this context, PMs are especially suited since these nanocarriers are able to incorporate both chemotherapeutic drugs and imaging agents, providing a theranostic platform for simultaneous diagnostic/imaging and therapeutic purposes in order to achieve customized and personalized cancer therapy that maximizes drug specificity and efficacy [28].

### Pegsitacianine

Pegsitacianine (ONM-100) is a pH-activable fluorescent PM made from amphiphilic block copolymers from the proprietary ON-BOARD™ polymeric micelle platform (OncoNano Medicine, Inc., Texas, USA), encapsulating indocyanine green (ICG), an FDA-approved NIR fluorophore [203]. The ON-BOARD™ platform uses UPS block copolymers based on PEG-*b*-PR, where PR is a hydrophobic polymer containing ionizable linear or cyclic tertiary amine groups/side chains [203]. The payloads can be physically entrapped or chemically bound to the polymeric micellar core, protected from systemic exposure [203].

The UPS block copolymers show positive cooperativity resulting from pH-triggered hydrophobic micellization above a sharp pH threshold while at lower pH values the neutral micelles dissociate into protonated dimers, releasing their payloads [204]. These binary on/off switchable systems with a transistor-like response to pH, which allow fine tuning of p*K*_a_ and pH transition sharpness by changing the hydrophobic chain length and/or alkyl substituents in the ionizable amino groups, provide useful probes for chemical and biological sensing [204].

The UPS nanoprobe pegsitacianine is an optimized pH-activatable ICG-encoded nanosensor (PINS) with a hydrodynamic diameter of 26.0 ± 1.1 nm, consisting of block copolymers of hydrophilic PEG and a hydrophobic block of ethylpropylaminoethyl (EPA) methacrylate and 2-aminoethyl methacrylate (AMA) random copolymers, p(EPA_100_-r-AMA-ICG), synthesized using the atom transfer radical polymerization (ATRP) method, and covalently conjugated to ICG via an amidation reaction between the AMA primary amine groups and the *N*-hydroxysuccinimide (NHS) ester derivative of ICG [203]. The PM is stable at physiological pH 7.4 and the fluorescence of ICG, sequestered within the hydrophobic segments of the micellar core, is quenched during circulation in the bloodstream due to homo-Förster resonance energy transfer (homo-FRET) between the dye molecules [203]. However, at pH 6.9 the cooperative dissociation of the micelles into the protonated unimers activates the fluorescent dye [203]. Thus, the nanoprobe allows the targeting of the acidic extracellular TME (pH 6.5–6.9), a metabolic hallmark common to most solid tumors regardless of their histology or anatomic location, avoiding the use of specific surface biomarkers with limited therapeutic utility due to genetic or phenotypic heterogeneity [203]. The pH nanotransistor, with binary off/on response at a transition pH of 6.9, amplifies the fluorescence signal in the tumor over that in the surrounding normal tissues, with suppression of signal in blood (pH 7.4) [203].

Pegsitacianine (2.5 mg/kg IV injection 24 h prior to imaging) was able to detect a broad range of human tumors (head and neck, breast, peritoneal metastasis, kidney, brain, and pancreatic tumors) in mouse models using existing clinical cameras [203]. Furthermore, pegsitacianine administration (2.5 mg/kg IV injection 12–24 h before surgery) to mice bearing human head and neck HN5 or triple-negative 4T1 breast orthotopic tumors allowed real-time tumor-acidosis-guided surgery (TAGS) of occult nodules (<1 mm^3^), significantly improving mice survival [203]. In murine flank xenograft models of lung cancer, the probe selectively labeled human adenocarcinoma (A549) as well as human squamous cell carcinoma (H1264) xenografts, with mean tumor-to-background ratio (TBR) > 2.0 for both histologic subtypes of lung cancer, according to NIR images 24 h after injection of 2 mg/kg pegsitacianine and further confirmed by microscopic analysis of tumor sections [205]. Pegsitacianine (1 mg/kg, IV) was administered to patients undergoing lung cancer surgery as part of an ongoing phase 2 trial (NCT05048082), now completed, and in the human pilot study the nanoprobe localized pulmonary adenocarcinoma (TBR 2.7) and pulmonary squamous cell carcinoma (TBR 2.4) in real time, corroborating successful clinical translation as an IMI probe to label human lung cancer [205].

A phase 1 clinical trial in the Netherlands (EudraCT 2017-003543-38, the SHINE study) exploiting metabolic acidosis in solid cancers using pegsitacianine for fluorescence-guided surgery reported that a 1.2 mg/kg single IV infusion of the nanoprobe 24 h ± 8 h prior to oncologic surgery was well tolerated and allowed visualization of four solid tumor types (HNSCC, breast cancer, esophageal cancer, and CRC) in 30 patients, both in vivo and ex vivo [206]. Among these patients, 13 subjects with HNSCC demonstrated that a fluorescent lesion on the surgical specimen with a TBR > 1.5 was correlated to a tumor-positive resection margin, detected directly after excision by fluorescence-guided intraoperative imaging [207].

During intraoperative fluorescence imaging, pegsitacianine enabled visualization of all the tumor-positive resection margins undetected during standard of care (SOC) surgery, which correlated with the final histopathological evaluation, yielding 100% sensitivity and no false negatives [206]. Moreover, additional occult lesions were identified via pegsitacianine fluorescence in five patients that would have been missed in SOC surgery [206]. This phase 1 study was the first to report a systemically administered probe displaying nanoscale cooperativity to overcome metabolic and phenotypic variability between different patients and tumors, with no overlap between tumor and background fluorescence [206].

In a non-randomized, open-label, phase 2 study (NCT04950166), administration of a single IV dose of pegsitacianine (1 mg/kg) to 50 patients 24–72 h before cytoreductive surgery and re-examination of the peritoneal cavity under NIR illumination following surgery allowed identification of eventual fluorescent tissue, which was excised and evaluated by histopathology [208]. Among the 40 patients evaluable for clinically significant events across six primary tumor types, residual disease was detected with pegsitacianine in 20 (50%) of them, and the high rate of occult residual disease detected suggests that the use of the probe can improve surgeon assessment and performance during cytoreductive surgery [208]. The probe showed high sensitivity and the absence of serious AEs, with only transient non-anaphylactic infusion reactions reported in 28% of patients [208]. Pegsitacianine has received Breakthrough Therapy Designation by the FDA as an adjunct for the visualization of metastatic disease in the peritoneal cavity in patients undergoing cytoreductive surgery in 2022 and is ready for phase 3 clinical evaluation.

## 7. Conclusions

Despite advancements in early detection and therapies, cancer remains a significant global health issue with a high incidence and mortality rate. Conventional chemotherapy is recognized as an effective and extensively used therapeutic option for most types of cancers. However, lack of selectivity, side effects, toxicity, and drug resistance are the main concerns, which can be potentially overcome by nanotechnology. Among nanoparticulate systems targeted to cancer cells, PMs stand out, efficiently encapsulating poorly water-soluble chemotherapeutic drugs, with their size, morphology, and solubility impacting circulation. They are simple to manufacture, increase the efficiency of drug loading, and can be easily customized and tailored to meet specific needs, which constitutes a distinct advantage over other nanoparticulate DDSs. PEGylation, decoration with targeting moieties, charge conversion, crosslinking of the core, and development of stimuli-responsive PMs have been employed to avoid MPS uptake, ameliorate pharmacokinetic properties, improve cellular internalization, and enhance selectivity of drug delivery towards cancer cells.

However, instability and a lack of economically viable production strategies restrict clinical translation, which will require better management of the partnership between the in vivo behavior and the physicochemical properties of PMs requiring an interplay between various domains of knowledge like chemistry, physics, biology, and nanomedicine. Nevertheless, several polymeric micellar formulations have entered clinical trials, and a few have obtained regulatory approval, such as Genexol^®^-PM and Nanoxel^®^, which have been in the clinic for almost twenty years.

Clinical failures of PMs may involve the payload, deficient tumor accumulation, the selection of individuals with diverse demographics, and more advanced tumor progression. Additionally, passive targeting by the EPR effect can differ significantly over time between patients and different tumor types, as well as within the same patient population, which could justify its publicized failure in the clinic. On the other hand, no animal model can accurately replicate all the features of human malignancy. Another major concern is the sustainable manufacture of high-quality clinical-grade PMs that adhere to Good Manufacturing Practice (GMP) standards, and the clinical safety issues related to toxicity.

Future directions for a successful translational from preclinical demonstrations to the multifunctional PMs loaded with anticancer drugs in the clinical setting may entail (i) stratification of patients; (ii) improved target-driven design; (iii) combined applications in the form of both multidrug nanomaterials and multimodal treatments; and (iv) advice from regulators who can identify any potential problems affecting the approval of the nanoformulation.

## Figures and Tables

**Table 2:** Classification of conventional (cytotoxic) chemotherapeutic drugs based on their mode of action.

Drug Class	Examples
Alkylating agents	Nitrogen mustards (bendamustine, chlorambucil, cyclophosphamide, ifosfamide, mechlorethamine, melphalan)
	Nitrosoureas (carmustine, lomustine, streptozocin)
	Platinum coordination complexes (cisplatin, carboplatin, oxaliplatin)
	Triazenes (dacarbazine, procarbazine, temozolomide)
	Alkyl sulfonate (busulfan)
	Ethyleneimine (thiotepa)
Antimetabolites	Folate antagonists (methotrexate, pemetrexed, pralatrexate, raltitrexed)
	Purine antagonists (fludarabine, 6-mercaptopurine, pentostatin, 6-thioguanine)
	Pyrimidine antagonists (azacitidine, capecitabine, cytarabine, 5-fluorouracil, gemcitabine)
Antibiotics	Anthracyclines (daunorubicin, doxorubicin, epirubicin, idarubicin), mitoxantrone
	Nonanthracyclines (bleomycin, dactinomycin, mitomycin)
Topoisomerase inhibitors	
Topoisomerase I inhibitors	Camptothecin derivatives (irinotecan, topotecan)
Topoisomerase II inhibitors - catalytic inhibitors - poisons	Epipodophylotoxins (etoposide, tenoposide) Anthracyclines (daunorubicin, doxorubicin, epirubicin, idarubicin), mitoxantrone
Mitotic inhibitors	
Microtubule-stabilizing agents	Taxanes (cabazitaxel, docetaxel, paclitaxel) Epothilones (Ixabepilone)
Microtubule-destabilizing agents	Vinca alkaloids (vinblastine, vincristine, vinorelbine)

**Table 4:** Polymeric micellar formulations for intravenous delivery of anticancer drugs or imaging agents in clinical trials. Data from US clinical trial website (https://clinicaltrials.gov, accessed on 12 June 2024).

Formulation	Drug (Combination Regimen)	Trial Identifier (Acronym)	Population (n)	Dose and Duration of Treatment	Phase	Start (Expected) Date–Completion (Estimated) Date (Status)
Polymeric micellar PTX	PTX (plus carboplatin and tislelizumab)	NCT06366945	Clinical N-positive HNSCC (n = 85)	PM-PTX (300 mg/m^2^), carboplatin (AUC 5), and tislelizumab (200 mg) every 3 weeks for 3 cycles	2	04/2024–05/2029 (not yet rec.)
Polymeric micellar PTX	PTX (plus cisplatin and capecitabin)	NCT06301165	Locoregionally advanced nasopharyngeal carcinoma (n = 162)	PM-PTX 200 mg/m^2^ on day 1, cisplatin 75 mg/m^2^ on day 1, and capecitabin 1000 mg/m^2^ on days 1–14 *versus* gemcitabine 1000 mg/m^2^ on days 1 and 8 and cisplatin 80 mg/m^2^ on day 1, every 3 weeks, for 3 cycles, followed by concurrent chemoradiotherapy (cisplatin 100 mg/m^2^, every 3 weeks, 3 cycles and IMRT)	2	03/2024–12/2028 (recruiting)
PTX polymer micelles	PTX (plus cisplatin and cadonilimab)	NCT06356688	Locally advanced esophageal squamous cell carcinoma (n = 30)	PM-PTX 230 mg/m^2^ infusion over 3 h in cycle 1 then 260 mg/m^2^ infusion over 3 h every 3 weeks in cycles 2–4, cisplatin 25 mg/m^2^/day (IV drip) for 3 days, every 3 weeks, and cadonilimab 375 mg (IV drip) on day 3, every 3 weeks	2	04/2024–06/2025 (not yet rec.)
Polymeric micellar PTX	PTX (plus cisplatin/carboplatin and sindilizumab)	NCT05782426	Advanced non-squamous NSCLC (n = 28)	PM-PTX, platinum (cisplatin/carboplatin) in combination with sindilizumab injection for 4–6 cycles; maintenance with sindilizumab plus PTX-PM injection (≤230 mg/m^2^)	2	03/2023–01/2026 (not yet rec.)
PTX polymeric micelles for injection	PTX	NCT06199895	Taxane-resistant pancreatic adenocarcinoma, cholangiocarcinoma, lung cancer, gastric cancer, esophageal carcinoma, or breast cancer (n = 25)	PM-PTX 300 mg/m^2^ infusion over 3 h once every 3 weeks (1 cycle treatment)	2	11/2023–11/2025 (recruiting)
PTX polymeric micelles for injection	PTX	NCT06143553	HER2-negative MBC (n = 168)	PM-PTX 300 mg/m^2^ infusion over 3 h, every 3 weeks (1 course treatment) versus albumin-bound PTX 260 mg/m^2^ infusion over 30 min, every 3 weeks	3	10/2023–07/2025 (recruiting)
DTX polymeric micelles for injection	DTX	NCT05254665	Advanced solid tumors (n = 110)	Dose confirmation stage (evaluation of safety and tolerability of 3 dosing regimens) followed by expansion stage (evaluation of efficacy and safety of best dosing regimen)	2	02/2022–03/2024 (unknown)
PTX micelles for injection	PTX	NCT04778839	Chinese patients with advanced solid tumors (n = 98)	PM-PTX (175, 260, 320, or 390 mg/m^2^ IV for 3 h) or conventional PTX injection (175 mg/m^2^ IV for 3 h), every 3 weeks	1	03/2021–03/2023 (recruiting)
PTX micelles for injection	PTX (plus cisplatin)	NCT02667743	First-line therapy for advanced NSCLC (n = 454)	PM-PTX (230 or 300 mg/m^2^ IV for 3 h) followed by cisplatin (70 mg/m^2^ IV for 2 h), every 3 weeks versus conventional PTX injection (175 mg/m^2^ IV for 3 h) followed by cisplatin (70 mg/m^2^ IV, for 2 h), every 3 weeks	3	05/2015–12/2021 (unknown)
Genexol^®^-PM	PTX	NCT03008512	Hepatocellular carcinoma after failure of sorafenib (n = 5)	Genexol^®^-PM 100 mg/m^2^ infusion for 1 h on days 1, 8, and 15 of a 28-day cycle, up to 8 cycles	2	10/2016–02/2021 (terminated due to poor accrual)
Genexol^®^-PM	PTX (plus gemcitabine)	NCT02739633	Recurrent/metastatic adenocarcinoma of the pancreas (n = 47)	Genexol^®^-PM (125 mg/m^2^ IV over 60 min) and gemcitabine (1000 mg/m^2^ IV) weekly, for 3 weeks, followed by 1 week of rest	2	04/2016–12/2019 (unknown)
Genexol^®^-PM	PTX (plus carboplatin)	NCT02739529	Gynecologic cancer (n = 18)	Genexol^®^-PM (100 or 120 mg/m^2^) IV infusion once a week and carboplatin (AUC 5 or 6) IV infusion every 3 weeks	1	04/2016–03/2018 (unknown)
Genexol^®^-PM	PTX (plus carboplatin)	NCT05300828	Ovarian cancer (n = 600)	Genexol^®^-PM (260 mg/m^2^, IV infusion over 3 h) followed by carboplatin (AUC 5, IV over 3 h), every 3 weeks, as adjuvant treatment after cytoreductive surgery	Obs.	10/2015–12/2022 (completed)
Genexol^®^-PM	PTX	NCT02064829 (TRIBECA)	Metastatic or locally recurrent breast cancer (n = 111)	Genexol^®^-PM or Nab-PTX, 260 mg/m^2^ infusion over 30 min every 3 weeks (bioequivalence study)	N/A	03/2014–07/2015 (completed)
Genexol^®^-PM	PTX (plus cisplatin)	NCT01689194	Locally advanced HNSCC (n = 53)	N/A	2	02/2013–08/2017 (completed)
Genexol^®^-PM	PTX (plus DOX)	NCT01784120	Advanced breast cancer (n = 48)	N/A	2	01/2011–12/2018 (unknown)
Genexol^®^-PM	PTX (plus gemcitabine)	NCT01770795	Advanced NSCLC (n = 45)	N/A	2	01/2011–10/2012 (completed)
Genexol^®^-PM	PTX (plus carboplatin)	NCT01276548	Ovarian cancer (n = 102)	Genexol^®^-PM (260 mg/m^2^, IV) or PTX (175 mg/m^2^, IV) plus carboplatin (5 AUC, IV)	2	10/2009–09/2012 (completed)
Genexol^®^-PM	PTX	NCT00912639	Taxane-pretreated recurrent breast cancer (n = 90)	Genexol^®^-PM (300 mg/m^2^ diluted in 500 mL of D5W or NS) infused for 3 h, every 3 weeks, for a minimum of 6 cycles	4	05/2009–05/2011 (unknown)
Genexol^®^-PM	PTX	NCT00876486	Recurrent or metastatic breast cancer (n = 213)	Genexol^®^-PM (260 mg/m^2^) or Genexol (175 mg/m^2^), IV infusion over 3 h, every 3 weeks	3	12/2008–11/2013 (completed)
Genexol^®^-PM	PTX (plus cisplatin)	NCT01023347	NSCLC (n = 276)	Genexol^®^-PM and cisplatin versus free PTX (Genexol^®^) and cisplatin	2	06/2008–06/2012 (completed)
Genexol^®^-PM	PTX (plus gemcitabine)	NCT00882973	Advanced pancreatic cancer (n = 18)	Genexol^®^-PM (220, 260, or 300 mg/m^2^) ad gemcitabine (1250 mg/m^2^)	1	09/2008–11/2010 (completed)
Genexol^®^-PM	PTX (plus carboplatin)	NCT00877253	Advanced ovarian cancer (n = 18)	Genexol^®^-PM (220, 260, or 300 mg/m^2^, IV infusion over 3 h) and carboplatin (AUC 5, IV infusion over 30–60 min), every 3 weeks	1	05/2008–06/2009 (completed)
Genexol^®^-PM	PTX	NCT01426126	Advanced urothelial cancer previously treated with gemcitabine and platinum (n = 37)	Genexol^®^-PM (240 mg/m^2^ diluted in 500 mL of D5W) IV infusion for 3 h every 3 weeks	2	12/2007–08/2011 (completed)
Genexol^®^-PM	PTX	NCT01169870	Anthracycline-pretreated MBC (n = 0)	Genexol^®^-PM (300 mg/m^2^ in 500 mL of D5W or NS, IV infusion over 1 h), every 3 weeks, up to 6 cycles versus PTX (175 mg/m^2^ in 500 mL of D5W or NS, IV infusion over 3 h), every 3 weeks	2	07/2007–10/2008 (withdrawn)
Genexol^®^-PM	PTX	NCT00111904	Unresectable locally advanced or metastatic pancreatic cancer (n = 43)	Genexol^®^-PM IV infusion over 3 h, every 3 weeks	2	05/2005–08/2007 (completed)
PTX-loaded polymeric micelle	PTX (plus carboplatin)	NCT00886717	Advanced ovarian cancer (n = 74)	Determine MTD and RP2D (phase 1) and evaluate the efficacy of the regimen in terms of CA-125 response rate after 6 courses of therapy (phase 2)	1/2	05/2008–n/a (unknown)
Nanoxel^®^	PTX (plus Herzuma^®^)	NCT03614364	Metastatic salivary duct carcinoma (n = 41)	Nanoxel^®^ 75 mg/m^2^ plus D5W 100 mL MIV over 1 h and trastuzumab-pkrb (Herzuma^®^) 8 mg/kg loading dose plus NS 250 mL MIV over 90 min or 6 mg/kg maintenance dose plus NS 250 mL MIV over 30 min, since cycle 2, every 3 weeks	2	09/2018–08/2023 (unknown)
Nanoxel^®^	PTX	NCT00915369	Advanced breast cancer (n = 24)	Nanoxel^®^ 220, 260, 310, or 375 mg/m^2^, up to 6 cycles	1	03/2009–04/2010 (unknown)
Nanoxel^®^ M	DTX, (plus DOX and cyclophosphamide	NCT05207514	Breast cancer (n = 26)	DOX (60 mg/m^2^, IV) and cyclophosphamide (600 mg/m^2^) followed by Nanoxel^®^ M (75 mg/m^2^) or Taxotere^®^ (75 mg/m^2^), every 3 weeks, for 4 cycles, as neoadjuvant chemotherapy	3	03/2022–05/2024 (terminated)
Nanoxel^®^ M	DTX	NCT04066335	NSCLC, breast, prostate, ovarian, head and neck, gastric, or esophageal cancers (n = 1498)	N/A	Obs.	09/2019–08/2024 (recruiting)
Nanoxel^®^ M	DTX (plus oxaliplatin)	NCT03585673 (DOSE)	Esophageal squamous cell carcinoma (n = 38)	Nanoxel^®^ M (35 mg/m^2^ IV over 1 h on days 1 and 8) and oxaliplatin (120 mg/m^2^ IV over 2 h on day 1), every 3 weeks	2	06/2018–03/2021 (unknown)
Nanoxel^®^ M	DTX	NCT02982395	BCG refractory non-muscle invasive bladder cancer (n = 36)	Nanoxel^®^M (75 mg in 100 mL NS, intravesical) or mitomycin (40 mg in 100 mL NS, intravesical)	3	01/2017–08/2018 (terminated)
Nanoxel^®^ M	DTX	NCT02639858	Recurrent or metastatic HNSCC (n = 31)	Nanoxel^®^ M (75 mg/m^2^ IV infusion)	2	10/2015–09/2020 (unknown)
NK-105	PTX	NCT01644890	Metastatic or recurrent breast cancer (n = 436)	NK-105 (65 mg/m^2^) or free PTX (80 mg/m^2^) on days 1, 8, and 15 of a 28-day cycle	3	07/2012–01/2017 (completed)
[89Zr]-Df-CriPec^®^ DTX	DTX	NCT03712423 (PICCOLO)	Immune-PET study in solid tumors (n = 7)	Low dose of [89Zr]-Df-CriPec^®^ DTX (0.1–2 mg) on day 1 followed by maximally 3 [89Zr] PET scans; 2 weeks later, unlabeled CriPec^®^ DTX (up to 60 mg/m^2^, IV, every 3 weeks) followed by a low dose of [89Zr]-Df-CriPec^®^ DTX and maximally 3 [89Zr] PET scans	1	04/2018–05/2020 (completed)
CriPec^®^ DTX	DTX	NCT03742713 (CINOVA)	Platinum resistant ovarian cancer (n = 25)	CriPec^®^ DTX (60 mg/m^2^ IV) every 3 weeks	2	10/2018–12/2020 (completed)
CriPec^®^ DTX	DTX	NCT02442531 (NAPOLY)	Solid tumors (n = 33)	CriPec^®^ DTX (dose escalation, start dose 15 mg/m^2^ IV), every 6 weeks, up to 6 cycles	1	08/2015–07/2018 (completed)
NK012	SN-38 (plus 5-FU)	NCT01238939	Solid tumors followed by dose expansion in patients with metastatic CRC (n = 35)	NK102 IV infusion over 30 min on day 1 and 5-FU IV continuous infusion (over 46 h) on days 1 and 15 of each 28-day cycle, for 6 cycles	1	08/2010–03/2014 (completed)
NK012	SN-38 (plus carboplatin)	NCT01238952	Solid tumors with dose expansion in triple-negative breast cancer (n = 4)	NK012 and carboplatin IV infusion once every 28 days	1	07/2010–03/2013 (completed)
NK012	SN-38	NCT00951054	Advanced, metastatic triple-negative breast cancer (n = 61)	NK102 (28 mg/m^2^ or 18 mg/m^2^ depending on UGT1A1 polymorphism) IV infusion over 30 min, once every 28 days	2	02/2009–02/2015 (completed)
NK012	SN-38	NCT00951613	Relapsed small cell lung cancer (n = 72)	NK102 (28 mg/m^2^ or 18 mg/m^2^ depending on UGT1A1 polymorphism) IV infusion over 30 min, once every 28 days	2	07/2009–01/2012 (completed)
NK012	SN-38	NCT00542958	Refractory solid tumors (n = 39)	NK012 dose escalating (9.0, 12.0, 16.0, 21.0, and 28.0 mg/m^2^) IV infusion over 30 min, every 3 weeks	1	03/2007–12/2011 (completed)
NC-6300	Epirubicin	NCT03168061	Advanced solid tumors or advanced, metastatic, or unresectable soft tissue sarcoma (n = 150)	NC-6300 IV infusion at escalating doses every 3 weeks (phase 1) followed by NC-6300 at the RP2D (phase 2)	1/2	06/2017–07/2020 (unknown)
HA132	Cisplatin	NCT05478785	Advanced malignant solid tumors (n = 126)	HA132 IV, every 3 weeks	1/2	08/2022–08/2025 (not yet recruit.)
NC-6004	Cisplatin (plus pembrolizumab)	NCT03771820	Recurrent or metastatic HNSCC that failed platinum or platinum-containing regimen (n = 136)	NC-6004 (90 mg/m^2^ up to 135 mg/m^2^ IV) followed by pembrolizumab (200 mg IV) infusion over 30 min, every 3 weeks	2	07/2019–04/2022 (unknown)
NC-6004	Cisplatin (plus 5-FU and cetuximab)	NCT03109158	Recurrent/metastatic HNSCC (n = 1)	Cetuximab followed by NC-6004 (RP2D established in Phase 1 and 5-FU	1/2	03/2017–03/2019 (completed)
NC-6004	Cisplatin (plus 5-FU and cetuximab)	NCT02817113	Recurrent and/or metastatic HNSCC (n = 4)	Cetuximab (400 mg/m^2^ IV infusion over 2 h on day 1 then 250 mg/m^2^ over 1 h, weekly) followed by NC-6004 (IV infusion over 1 h) on day 1, every 3 weeks, and 5-FU (1000 mg/m^2^/day) on days 1–4 as continuous infusion, every 3 weeks	1	06/2016–09/2018 (terminated, strategy changed)
NC-6004	Cisplatin (plus gemcitabine)	NCT02240238	Advanced solid tumors or NSCLC, biliary tract, and bladder cancers (n = 209)	NC-6004 at escalating doses (60, 75, 90, 105, 120, 135, 150, 165, or 180 mg/m^2^) on day 1 followed by gemcitabine (1250 mg/m^2^) IV infusion over 30 min on days 1 and 8 of each cycle	1b/2	05/2015–05/2019 (completed)
NC-6004	Cisplatin (plus gemcitabine)	NCT02043288	Locally advanced or metastatic pancreatic cancer (n = 310)	NC-6004 90 mg/m^2^ IV infusion over 60 min on day 1 and gemcitabine 1000 mg/m^2^ IV infusion over 30 min on days 1 and 8, for 3-week cycle, or gemcitabine alone (1000 mg/m^2^ IV infusion over 30 min on days 1, 8, and 15, for 4-week cycle)	3	01/2014–12/2019 (completed)
NC-6004	Cisplatin (plus gemcitabine)	NCT00910741	Locally advanced and metastatic pancreatic cancer (n = 40)	NC-6004 once every 3 weeks followed by gemcitabine on days 1 and 8 of the 3-week cycle	1/2	05/2009–07/2013 (completed)
NC-4016	DACHPt	NCT03168035	Advanced solid tumors or lymphoma (n = 34)	NC-4016 dose escalating (15, 25, 30, 40, 60, and 80 mg/m^2^ IV infusion over 2 h), once every 3 weeks	1	11/2013–04/2017 (completed)
ONM-501	cGAMP	NCT06022029	Advanced solid tumors and lymphomas (n = 168)	ONM-501 intratumoral injection weekly, for 3 weeks, followed by a 3-week interval, w/o cemiplimab (Libtayo^®^) 350 mg IV infusion every 3 weeks	1	10/2023–08/2026 (recruiting)
Pegsitacianine	ICG	NCT05048082	Detection of lung malignancies in patients undergoing routine surgery (n = 24)	Pegsitacianine (1 mg/mL single dose, IV) infused 24–72 h prior to surgery	2	04/2022–08/2022 (completed)
Pegsitacianine	ICG	NCT04950166	Detection of peritoneal metastases in patients undergoing cytoreductive surgery (n = 50)	Pegsitacianine (1 mg/mL single dose, IV) infused 24–72 h prior to surgery	2	11/2021–01/2023 (completed)
Pegsitacianine	ICG	NCT03735680	Cancer detection in patients with solid tumors (n = 30)	Pegsitacianine single dose (1.0, 2.0, or 3.0 mg/kg) before surgery and fluorescence imaging at 3 ± 2 h, 6 ± 3 h, or 24 ± 8 h postdose	2a	08/2019–11/2021 (completed)

Abbreviations: AUC, area under the plasma concentration–time curve; BCG, Bacillus Calmette–Guérin; CA-125, cancer antigen 125; cGAMP, cyclic guanosine monophosphate (GMP)-adenosine monophosphate (AMP); CRC, colorectal cancer; D5W, dextrose 5% in water; DACHPt, dichloro(1,2-diaminocyclohexane)platinum(II); DOX, doxorubicin; DTX, docetaxel; 5-FU, 5-fluorouracil; HNSCC, head and neck squamous cell carcinoma; ICG, indocyanine green; IMRT, intensity-modulated radiation therapy; IV, intravenous; MBC, metastatic breast cancer; MIV, multiple intravenous (infusions); MTD, maximum tolerated dose; N/A, not available; Nab, nanoparticle albumin-bound; NS, normal saline; NSCLC, non-small cell lung cancer; PET, positron emission tomography; PM, polymeric micelle; PTX, paclitaxel; RP2D, recommended phase 2 dose; SN-38, 7-ethyl-10-hydroxycamptothecin; UGT1A1, uridine diphosphate (UDP)-glucuronosyltransferase 1A1.

## Data Availability

Not applicable.
